# Three Contactless Sleep Technologies Compared With Actigraphy and Polysomnography in a Heterogeneous Group of Older Men and Women in a Model of Mild Sleep Disturbance: Sleep Laboratory Study

**DOI:** 10.2196/46338

**Published:** 2023-10-25

**Authors:** Kiran K G Ravindran, Ciro della Monica, Giuseppe Atzori, Damion Lambert, Hana Hassanin, Victoria Revell, Derk-Jan Dijk

**Affiliations:** 1 Surrey Sleep Research Centre, School of Biosciences, Faculty of Health and Medical Sciences University of Surrey Guildford United Kingdom; 2 UK Dementia Research Institute, Care Research and Technology Centre at Imperial College, London and the University of Surrey Guildford United Kingdom; 3 Surrey Clinical Research Facility, School of Biosciences, Faculty of Health and Medical Sciences Guildford United Kingdom; 4 National Institute for Health Research - Royal Surrey Clinical Research Facility Guildford United Kingdom

**Keywords:** contactless sleep technologies, evaluation, nearables, polysomnography, older adults, sleep, Withings sleep analyzer, Emfit, Somnofy

## Abstract

**Background:**

Contactless sleep technologies (CSTs) hold promise for longitudinal, unobtrusive sleep monitoring in the community and at scale. They may be particularly useful in older populations wherein sleep disturbance, which may be indicative of the deterioration of physical and mental health, is highly prevalent. However, few CSTs have been evaluated in older people.

**Objective:**

This study evaluated the performance of 3 CSTs compared to polysomnography (PSG) and actigraphy in an older population.

**Methods:**

Overall, 35 older men and women (age: mean 70.8, SD 4.9 y; women: n=14, 40%), several of whom had comorbidities, including sleep apnea, participated in the study. Sleep was recorded simultaneously using a bedside radar (Somnofy [Vital Things]: n=17), 2 undermattress devices (Withings sleep analyzer [WSA; Withings Inc]: n=35; Emfit-QS [Emfit; Emfit Ltd]: n=17), PSG (n=35), and actigraphy (Actiwatch Spectrum [Philips Respironics]: n=18) during the first night in a 10-hour time-in-bed protocol conducted in a sleep laboratory. The devices were evaluated through performance metrics for summary measures and epoch-by-epoch classification. PSG served as the gold standard.

**Results:**

The protocol induced mild sleep disturbance with a mean sleep efficiency (SEFF) of 70.9% (SD 10.4%; range 52.27%-92.60%). All 3 CSTs overestimated the total sleep time (TST; bias: >90 min) and SEFF (bias: >13%) and underestimated wake after sleep onset (bias: >50 min). Sleep onset latency was accurately detected by the bedside radar (bias: <6 min) but overestimated by the undermattress devices (bias: >16 min). CSTs did not perform as well as actigraphy in estimating the all-night sleep summary measures. In an epoch-by-epoch concordance analysis, the bedside radar performed better in discriminating sleep versus wake (Matthew correlation coefficient [MCC]: mean 0.63, SD 0.12, 95% CI 0.57-0.69) than the undermattress devices (MCC of WSA: mean 0.41, SD 0.15, 95% CI 0.36-0.46; MCC of Emfit: mean 0.35, SD 0.16, 95% CI 0.26-0.43). The accuracy of identifying rapid eye movement and light sleep was poor across all CSTs, whereas deep sleep (ie, slow wave sleep) was predicted with moderate accuracy (MCC: >0.45) by both Somnofy and WSA. The deep sleep duration estimates of Somnofy correlated (*r*^2^=0.60; *P*<.01) with electroencephalography slow wave activity (0.75-4.5 Hz) derived from PSG, whereas for the undermattress devices, this correlation was not significant (WSA: *r*^2^=0.0096, *P*=.58; Emfit: *r*^2^=0.11, *P*=.21).

**Conclusions:**

These CSTs overestimated the TST, and sleep stage prediction was unsatisfactory in this group of older people in whom SEFF was relatively low. Although it was previously shown that CSTs provide useful information on bed occupancy, which may be useful for particular use cases, the performance of these CSTs with respect to the TST and sleep stage estimation requires improvement before they can serve as an alternative to PSG in estimating most sleep variables in older individuals.

## Introduction

### Background

Sleep is a major determinant of the quality of life, and sleep disturbances are a risk factor for a variety of health conditions. Longitudinal monitoring of objective sleep measures using unobtrusive, low-cost technologies will allow data-driven identification of clinical biomarkers and covariates of sleep and health and facilitate better monitoring of the efficacy of sleep interventions [1,2]. This is particularly relevant in aging, as sleep disturbances increase with age and are a risk factor for several health conditions prevalent in older adults, including dementia.

Polysomnography (PSG) is considered the gold standard for evaluating sleep, but longitudinal implementation of PSG at scale is not feasible because of the cost and burden it imposes on the user. Rest-activity monitoring (actigraphy) through a wrist-worn wearable device is currently the most widely used alternative for monitoring sleep in real-world settings. However, actigraphy is limited to a binary classification (sleep vs wake) and underestimates wake, especially in disrupted sleep and sleep disorders [3]. Furthermore, similar to PSG, clinical-grade actigraphy devices are of relatively high cost.

Consumer-grade low-cost wearable devices (wearables) are a potential alternative to clinical-grade actigraphy devices. However, wearables are not an ideal solution for longitudinal monitoring because they still pose a burden to the user and rely on portable battery technology (need to be recharged); therefore, their acceptability, especially in older people and people living with dementia, may be low [4-7].

### Contactless Sleep Technology

Contactless sleep technologies (CSTs), also known as nearables, are of great interest for conducting longitudinal sleep recordings in general and in older individuals and those with mild cognitive impairment or dementia in particular. This is because, unlike wearables, CSTs do not have to be worn, are inconspicuous (embedded into the living environment), do not impose any burden on the user, and are not constrained by limited battery life because they are wired to the mains electricity. Furthermore, CSTs are designed within the context of digital platforms that allow data to be collected, relayed to cloud storage facilities, and processed to provide objective sleep and vital sign measures. With CSTs demonstrating acceptable accuracy compared with the existing gold standards, they could play an integral part in creating a “bedroom of the future,” enabling long-term, continuous, and real-time remote monitoring of sleep and physiology at night by clinicians and health care providers. They may also support and monitor the effectiveness of interventions for improving sleep, which may ultimately facilitate longer independent living for older adults [8,9].

Bedside radars and undermattress devices are the 2 most commonly used types of CSTs. Bedside radars use radar technology (commonly ultrawideband radio frequency), whereas the various undermattress devices use several sensing technologies, such as pneumatic, piezoelectric, and electromechanical films. Although contactless devices use a variety of sensing approaches, the main information acquired by these devices is based on the ballistographic signal. This is a composite signal containing a wealth of information on body movements, breathing, and cardiac activity, which can be used to estimate wake and sleep stages [10].

The plethora of low-cost CSTs opens up a wide range of possibilities for longitudinal sleep monitoring at scale in home settings. However, only a handful of these consumer-grade devices have been evaluated against PSG or actigraphy [8,11]. Without rigorous evaluation, there are also potential risks of the multimodal capabilities and clinical value of CSTs not being used to their full potential and the data being misinterpreted [12]. Several of these validation studies have important limitations. CST sleep prediction algorithms are usually trained on healthy participants, which limits the heterogeneity of the training data and potentially affects real-world performance in more heterogeneous populations. Most studies that intend to validate these devices and their algorithms, which are preferably referred to as evaluation studies rather than validation studies [13], are conducted in young healthy adults without sleep disturbances rather than in older participants with health problems and sleep disturbance. Furthermore, most evaluation studies do not offer interdevice comparisons, thus limiting conclusions on the relative performance of the devices [14-17]. Another important but often overlooked issue in evaluating CSTs is the consideration of the period over which “sleep” is analyzed. In PSG studies conducted in sleep laboratories, the analysis period (AP) is defined as the interval between lights off and lights on. For actigraphy studies at home, the American Academy of Sleep Medicine (AASM) [18] recommendation is that the AP be derived from a sleep diary [19]. The sleep measures are then estimated using this AP. By contrast, consumer sleep technologies rely on the automatic estimation of the rest (or time-in-bed) period. In many evaluation studies, the AP is nevertheless set to the AP according to the PSG, but this may yield biased performance metrics that are not relevant to the use of these devices in the real world. A final consideration for evaluation studies is the choice of performance measures. Traditional measures such as sensitivity and accuracy may not be appropriate in cases in which there are imbalances in the number of observations across classes, such as in sleep-wake classification [20,21].

Evaluating CSTs against gold-standard PSG and actigraphy in older adults to understand their accuracy and reliability requires evaluation protocols that address the above-discussed issues. We have previously shown that CSTs provide accurate information on “bed occupancy” in older people in a home setting [22]. Here, the performance of 3 contactless devices, namely a bedside radar (Somnofy [Vital Things]) and 2 undermattress devices (Withings sleep analyzer [WSA; Withings Inc] and Emfit-QS [Emfit; Emfit Ltd]), in estimating sleep parameters was evaluated in comparison with those of gold-standard PSG and actigraphy (Actiwatch Spectrum [AWS; Philips Respironics]) in a heterogeneous population of older men and women. We made use of the “first night effect,” which refers to the reduced quality of sleep when participants are sleeping in a novel environment [23]. In addition, an extended (10-hour) time-in-bed period was imposed to better mimic sleep patterns in dementia. We addressed the pitfalls in evaluating CSTs and quantified the agreement between these CSTs and PSG and between these CSTs and actigraphy (AWS) using different performance measures and AP definitions. The usefulness of CSTs may vary across use cases. For some use cases, a simple all-night estimate of the total sleep time (TST) may be sufficient, whereas for other use cases, it is necessary to estimate sleep stages and epoch-by-epoch (EBE) concordance. The performance of these devices was, therefore, evaluated both with respect to all-night summary measures or EBE concordance and at various levels of characterization of the sleep-wake phenotype, that is, from a simple 2 category classification, namely wakefulness and sleep, to 4 category classification, namely wake, light sleep (LS), deep sleep (DS), and rapid eye movement (REM) sleep.

## Methods

### Study Population

Participants were recruited to the study via a targeted search of the Surrey Clinical Research Facility participant database, followed by telephone screening and self-reported assessment of health. During an in-person screening visit, an array of assessments and clinical procedures was performed to determine suitability for the study. Participants were considered eligible if they met the inclusion criteria (being aged 65 to 85 y, living independently, being a nonsmoker, having self-declared stable medical conditions, and consuming <28 units of alcohol per wk). Data were collected from 2 cohorts: cohort 1 with 18 participants (January to March 2020) and cohort 2 with 17 participants (June to November 2021).

### Study Protocol

Participants came to the sleep laboratory (Surrey Sleep Research Centre, Guildford, United Kingdom) for 1 overnight sleep recording after they had been using the devices for 8 to 14 days at home (the analyses of the home data have been reported elsewhere [22]). We deliberately included no adaptation night in this protocol and used the first night effect to create conditions of mild sleep disturbance [23]. During the overnight in-laboratory study, participants were provided with a time-in-bed period of 10 hours. Full PSG was recorded according to the AASM guidelines using the SomnoHD PSG system (SOMNOmedics GmbH). Sleep (sleep stages: wake, REM, stage N1 of non-REM sleep [N1], stage N2 of non-REM sleep [N2], and stage N3 of non-REM sleep [N3]) was scored at 30-second intervals in the DOMINO software environment (SOMNOmedics GmbH) by 2 independent scorers, and a consensus hypnogram was generated. All recordings were visually inspected, and artifacts were removed. We estimated 30-second epoch-wise slow wave activity (SWA) power (0.75-4.5 Hz) from the electroencephalography (EEG) spectrogram created by the fast Fourier transform applied to 4-second epochs after tapering with a hamming window. In general, the left frontal referenced to right mastoid (F3-M2) channel derivation was used for the computation of SWA unless the quality of this channel was poor, in which case the right frontal referenced to left mastoid (F4-M1) channel derivation was used.

The PSG sleep summary estimates were computed for the interval from lights off to lights on, as per the AASM guidelines. The apnea hypopnea index (AHI) was obtained by applying >3% oxygen desaturation or a respiratory event accompanied by an arousal as a criterion for identifying hypopnea [18]. Other relevant study population measures such as the Mini-Mental State Examination, Pittsburgh Sleep Quality Index (PSQI), and Epworth Sleepiness Scale (ESS) were collected using questionnaires.

### Contactless Sleep Trackers and Actigraphy Device Evaluated

This study was conducted in 2 cohorts. In cohort 1 (n=18), we evaluated the WSA and AWS. The devices evaluated in cohort 2 (n=17) were the WSA, Emfit, and Somnofy bedside radar. The contactless device and AWS data were simultaneously collected along with PSG. Time synchronization was assessed and achieved across the devices (including PSG) by connecting them to the same secure network.

The WSA is a pneumatic undermattress device, and the Emfit is an electromechanical film undermattress device. Both devices were placed underneath the mattress and adjacent to each other at the thoracic level [16,24]. The bedside radar (Somnofy) device uses low-power ultrawideband radar and was placed on a bedside table pointing toward the participant’s thorax [15]. The devices were set up as per the manufacturer’s guidelines. All 3 devices detect activity and physiological parameters to identify sleep stages. The bedside radar (Somnofy) is the only device in the study that performs active sensing using radio waves (via the Doppler radar technique) and collects environmental variables such as light, sound, and particulate pollution.

AWS is a standard clinical-grade actigraphy device that has been widely deployed for home monitoring and evaluated against PSG in the sleep literature [25,26]. Actiware (version 6.0.7; Philips Respironics) was used to set up the device and download and analyze the collected data. AWS was set up to output sleep and activity labels at 60-second epoch and synchronized to the clock time by the Actiware during configuration. The device estimates the wearer’s activity in terms of activity counts per epoch and assigns sleep or wake labels using a preset threshold. The activity threshold was set to medium threshold (epochs with an activity count over 40 were labeled by the device as wake) [22].

### Collected Data

The WSA and Somnofy data were downloaded using the application programming interface provided by the respective manufacturer, whereas the Emfit device data were downloaded from a simple web interface. The Emfit data consisted of “csv” files, whereas the WSA and Somnofy data consisted of “json” files. A further description of device deployment is provided in *Contactless Sleep Technology* section and Table S1 in Multimedia Appendix 1.

The contactless devices generated a 4-stage hypnogram with DS (assumed to be equivalent to N3 sleep), LS (assumed to be equivalent to N1 or N2 sleep), REM, and wake labels. The temporal resolution of the hypnograms generated by Emfit and Somnofy was 30 seconds, whereas for WSA and AWS, the temporal resolution was 60 seconds. Because the analysis was performed on clock time, we made the relevant daylight-saving correction to the device Coordinated Universal Time series. The Emfit and Somnofy time series were further synchronized to PSG using the activity or movement data from the respective devices through cross-correlation [15]. PSG was available for all the participants, but owing to the differences in device deployment between cohorts 1 and 2, erroneous automated summary, and data loss, the number of nights differed between devices. The total number of nights of data used for each device was as follows: WSA: n=35 (34 for the automated device-selected AP), AWS: n=18, Somnofy: n=17, and Emfit: n=16.

All the 3 contactless devices automatically detected the overnight in-bed periods and generated sleep-wake summary estimates for these periods, including TST, sleep onset latency (SOL), wake after sleep onset (WASO), sleep efficiency (SEFF), and sleep stage (DS, LS, REM, and wake) duration estimates. We combined the LS and DS estimates to derive the non-REM (NREM) duration. The AWS data were downloaded, and sleep-wake time series and sleep summary measures were generated using the Actiware. The APs or rest intervals for sleep summary generation can be determined automatically by the Actiware or set manually by the user.

### Performance Assessment Categories and APs

Performance analysis can be broadly separated into 2 categories: sleep summary measures, which provide the cumulative estimates of nocturnal sleep, and EBE concordance, resulting in detailed information on the sleep architecture detection accuracy of the devices under evaluation.

All the evaluated CSTs detected the overnight-in-bed periods based on their respective proprietary algorithms and generated sleep or wake summary measures. The sleep summary estimates associated with this automated device-selected AP are referred to as the “analysis period–automatic (AP-A).” Further, the sleep summary estimates of the devices were calculated using the sleep stage time series during the period from lights off to lights on (referred to as “analysis period–manual [AP-M]”). AP-M allowed us to compare the devices against PSG for the same AP. For the sleep summary agreement evaluation, the above-mentioned AP-A and AP-M estimates were compared with PSG sleep summary estimates.

EBE agreement analysis was performed for the total recording interval of PSG (from the start of PSG recording to the end of PSG recording) and for the period from lights off to lights on for completeness. The performance of each device was compared with that of the gold-standard PSG for all available nights. All the data analyses reported here were performed using MATLAB (version 2021b; Math Works).

To determine the satisfactory level of agreement in sleep summary estimates and EBE concordance between the device and PSG estimates, we used the interscorer difference estimates available in the literature. The metrics used and their agreement thresholds are discussed in the following sections.

The number of participants for whom data were available was not the same for all the CSTs owing to the varied deployment in cohorts 1 and 2 and errors in data collection. For each comparison, the maximum number of available participants was used for performance assessment.

### Sleep Summary Agreement Assessment Approach

The sleep or wake summary measures available across all devices were TST (total time spent in sleep during the “lights off” period as assessed by PSG), SOL (time elapsed from lights off to the first incidence of sleep), WASO (time spent in wake during the “lights off” period as assessed by PSG), and SEFF (ratio of the TST to the total recording time (TRT) of PSG expressed as percentage). Hence, these summary measures were used for comparisons across all the devices and APs (AP-A and AP-M). In addition, for the contactless devices, but not AWS, sleep stage duration measures such as LS, DS, REM sleep, and wake durations were also compared. The results of the AP-M analysis are presented in Multimedia Appendix 1. Bland-Altman plots were created to understand the agreement between the device-estimated measures and the gold-standard PSG measures. The Shapiro-Wilk test was performed to check the normality of the differences [27-30]. We found that for most sleep parameters, the differences passed the normality test. The exceptions were the AP of AWS automatic analysis and Somnofy and SOL of Somnofy. These deviations from normality were deemed to be related to the small sample size and outliers, and no corrections were made.

Apart from the bias, limits of agreement, and the associated 95% CIs, we estimated the minimum detectable change and Pearson correlation (ρ) and assessed the reliability using consistency intraclass correlation (ICC) with 2-way random effects, and effect size for the magnitude of differences (Cohen *d* or standardized difference here) [27].

Sleep summary measures have values ranging from few minutes to hundreds of minutes (SEFF is a percentage measure). Owing to this difference in measurement range and units across the different sleep summary measures, the above-mentioned traditional metrics do not allow direct intermetric comparison across the different devices or the ranking of device performance. To address this problem, we used standardized metrics such as the symmetric mean absolute percentage error (SMAPE) [31] and standardized absolute difference (SAD) to quantify the bias and dispersion in different sleep measures (sleep-wake and sleep stage duration). Given that *x* is the reference device estimate (PSG) and *y* is the test device estimate with *n* simultaneous measurements, SMAPE and SAD are defined as follows:



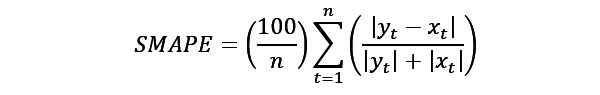

**(1)**




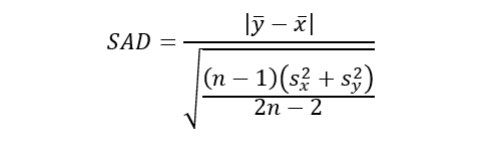

**(2)**


Here, *s^2^* is the variance of the reference and test device measurements. These measures are directionless and unitless and hence allow for direct comparisons of the measurement agreement across devices and estimates. Standardized differences, Cohen *d*, and SAD were classified as follows: 0.1-<0.3=small; 0.3-<0.5=moderate; ≥0.5=large. SMAPE values ranged from 0% to 100%.

The estimates of the average agreement (ICC) between scorers and the average score reported by Younes et al [32] were used to define the satisfactory agreement level for the sleep summary measures. The ICC thresholds for the different duration estimates were as follows: 0.84 for WASO, 0.75 for REM, 0.65 for NREM, 0.67 for LS; and 0.63 for DS.

### EBE Concordance Assessment Approach

The concordance between the sleep stage hypnogram time series automatically generated by each device and the PSG hypnogram was estimated for the total recording interval of PSG. The 5-stage PSG hypnogram was converted into a 4-stage hypnogram similar to the device hypnograms by combining N1 and N2 as LS (LS=N1 or N2) and assuming N3 as DS. The concordance analysis was performed at the level of PSG hypnogram resolution, which was in 30-second intervals. The 60-second WSA and AWS hypnograms were converted to 30-second resolution by imputing the unavailable 30-second epoch data with the label of the next adjacent minute. Epochs scored as artifacts in PSG and missing (WSA) and no presence (Somnofy and Emfit) epochs in the devices were excluded from the concordance analysis, that is, only valid or complete pairs of hypnogram labels between PSG and the devices were used for the analysis. Finally, to achieve an accurate EBE concordance assessment, PSG, AWS, and CST hypnograms were aligned via cross-correlation, and the lag within a 10-epoch window that provided the best alignment and concordance was used.

To investigate the changes in concordance with different sleep staging resolutions, the analysis was performed at the following three levels of the hypnogram: (1) two stages (sleep and wake), (2) three stages (NREM [LS or DS], REM, and wake), and (3) four stages (DS, LS, REM, and wake). In addition, we performed EBE concordance analysis for the lights off period (Multimedia Appendix 1). Sensitivity (sleep prediction accuracy) and specificity (wake prediction accuracy) are reported for sleep or wake EBE analysis. For the different sleep stage concordance analysis, sensitivity, specificity, accuracy, *F*_1_-score, and Matthew correlation coefficient (MCC, which accounts for class imbalance and is a better alternative to the κ metric or its variants) are reported for completeness and consistency with the existing literature [20,21].

The estimates of the interrater reliability reported by Lee et al [33] were used to define the satisfactory agreement level for the EBE concordance. Because MCC and the κ metrics have almost identical values when both metrics are in the positive quadrant [20], we used the κ values reported by Lee et al [33] to define the MCC threshold for satisfactory EBE concordance. The MCC thresholds for the different sleep stages were as follows: 0.70 for sleep or wake, 0.69 for REM, 0.48 for NREM, 0.40 for LS, and 0.57 for DS.

### Ethical Considerations

The study received a favorable opinion from the University of Surrey Ethics Committee (reference UEC 2019 065 FHMS) and was conducted in accordance with the Declaration of Helsinki, the Principles of Good Clinical Practice, and relevant guidelines and regulations of the University of Surrey. Potential participants were given detailed information about the study protocol, and they provided written informed consent before any study procedures were performed.

## Results

### Study Population Characteristics

The study involved a total of 35 participants (age: mean 70.8, SD 4.86; range 65-83 y; women: n=14, 40%; men: n=21, 60%) with no self-reported history of mental health or neurological problems. Cohorts 1 and 2 were similar with respect to demographics and PSG-assessed sleep parameters (Table 1). Approximately 60% (20/35) of the participants had ≥1 self-reported comorbidities, including type-2 diabetes (2/35, 6%), hypertension (2/35, 6%), obesity (BMI>30; 6/35, 17%), and arthritis (6/35, 17%). Reported comorbidities were stable and well controlled, with no recent medication changes or hospitalizations that interfered with the study conduct.

The values shown in Table 1 are mean, SD, and range. The significance of the difference between cohorts 1 and 2 is given along with the effect size (Cohen *d*). Significant differences (*P*<.05) are highlighted in italics. AP in PSG is the period from the lights off to lights on (AP-M).

None of the participants were below the cutoff (23) for clinically significant cognitive impairment as indexed by the Mini-Mental State Examination. The PSQI scores were on average <5, with the highest score being 10, which indicates that the majority of the participants did not have clinically significant sleep disturbance (PSQI>5). None of the participants experienced excessive daytime sleepiness as indexed by the ESS (>10). Nevertheless, the clinical PSG recording revealed that 49% (17/35) of the participants had moderate (9/35, 26%; AHI: 15 to <30) or severe (8/35, 23%; AHI: >30) sleep apnea, whereas 46% (16/35) of the participants had mild apnea (AHI: 5 to <15). In view of the health status of the participants, we refer to this study population as “heterogeneous.”

The sleep opportunity period (period available for sleep, described here as the AP), set to “lights off” period as per AASM guidelines [18], ranged from 466 to 586 minutes. TST ranged from 282 to 504 minutes, leading to sleep efficiencies ranging from 52.7% to 92.6%. SOL was, on average, within a normal range (normal reference SOL [age 65-79 y]: mean 19.5, 95% CI 15.2-23.8 min [34]), with the longest SOL being 49.5 minutes. All sleep stages were present in all participants. REM sleep duration was relatively short, and WASO was high, with considerable between-participant variation. The definition of the PSG sleep summary measures and a detailed summary of the device sleep summary characteristics of the participants can be found in Tables S2 and S3 in Multimedia Appendix 1.

**Table 1:** Study population characteristics.

Characteristics		Cohort 1 (n=18), mean (SD; range)	Cohort 2 (n=17), mean (SD; range)	*P* value	Effect size	Pooled (n=35), mean (SD; range)
**Demographics**						
	Age (y)	69.67 (5.04; 65-80)	72 (4.49; 65-83)	.16	−0.50	70.8 (4.86; 65-83)
	Gender (women), n (%)	8 (44)	6 (35)	N/A^a^	N/A	14 (40)
	BMI (kg/m^2^)	27.03 (4.84; 21.87-39.75)	26.42 (4.71; 20-36.8)	.71	0.13	26.73 (4.72; 20-39.75)
	AHI^b^ (events/h)	20.89 (17.46; 1.6-66.7)	18.96 (13.62; 4.2-58.8)	.72	0.13	19.95 (15.51; 1.6-66.7)
	MMSE^c^	28.44 (1.46; 25-30)	28.88 (1.32; 25-30)	.36	−0.32	28.66 (1.39; 25-30)
	PSQI^d^	4.22 (1.86: 1-7)	4 (2.35; 1-10)	.76	0.11	4.11 (2.08; 1-10)
	ESS^e^	3.72 (2.67; 1-9)	3.47 (2.32; 0-8)	.77	0.10	3.6 (2.47; 0-9)
**Polysomnography sleep measures**						
	TRT^f^ (min)	600.03 (1.54; 596.11-603.35)	601.2 (9.95; 590.25-622.43)	.62	−0.17	600.6 (6.93; 590.25-622.43)
	AP^g^ (min)	571.75 (18.49; 523.78-586)	512.39 (22.79; 466-539.89)	*.001* ^h^	*2.95*	542.91 (36.36; 466-586)
	TST^i^ (min)	422.47 (53.16; 326.5-504)	347.32 (55.46; 282-469)	*.001*	*2.95*	385.97 (65.67; 282-504)
	SOL^j^ (min)	11.56 (10.62; 0-40.5)	18.91 (13.71; 2-49.5)	.08	−0.62	15.13 (12.60; 0-49.5)
	WASO^k^ (min)	130.44 (47.56; 60-206.5)	146.09 (56.43; 30.5-240)	.38	−0.31	138.04 (51.89; 30.5-240)
	SEFF^l^ (%)	73.86 (8.82; 57.28-87.56)	67.92 (11.33; 52.27-92.6)	.09	0.60	70.99 (10.41; 52.27-92.6)
	N3^m^ (percentage of TST)	20.21 (5.94; 11.58-36.48)	19.74 (9.66; 3.08-34.93)	.86	0.06	19.98 (7.85; 3.08-36.48)
	N2^n^ (percentage of TST)	47.70 (8.08; 33.69-61.55)	47.27 (7.83; 36.5-61.1)	.87	0.05	47.49 (7.85; 33.69-61.55)
	N1^o^ (percentage of TST)	17.08 (7.12; 5.58-37.8)	19.22 (9; 7.46-36.89)	.001	−4.15	18.12 (8.04; 5.58-37.81)
	REM^p^ (percentage of TST)	15 (5.99; 5.49-23.96)	13.77 (4.93; 3.39-19.65)	.51	0.23	14.41 (5.46; 3.39-23.96)
	NREM^q^ (percentage of TST)	84.99 (5.99; 76.04-94.51)	86.23 (4.93; 80.35-96.6)	.52	−0.23	85.59 (5.46; 76.04-96.6)
	Wake (min)	142 (50.83; 62-243.5)	165 (60.12; 37.5-257.5)	.23	−0.43	153.17 (55.94; 37.5-257.5)

^a^N/A: not applicable.

^b^AHI: apnea hypopnea index.

^c^MMSE: Mini-Mental State Examination.

^d^PSQI: Pittsburgh Sleep Quality Index.

^e^ESS: Epworth Sleepiness Scale.

^f^TRT: total recording time.

^g^AP: analysis period.

^h^Significant differences (*P*<.05) are italicized.

^i^TST: total sleep time.

^j^SOL: sleep onset latency.

^k^WASO: wake after sleep onset.

^l^SEFF: sleep efficiency.

^m^N3: stage N3 of non–rapid eye movement sleep.

^n^N2: stage N2 of non–rapid eye movement sleep.

^o^N1: stage N1 of non–rapid eye movement sleep.

^p^REM: rapid eye movement.

^q^NREM: non–rapid eye movement.

### Examples of All-Night Recordings Obtained via PSG, AWS, and CSTs

**Figure 1:**
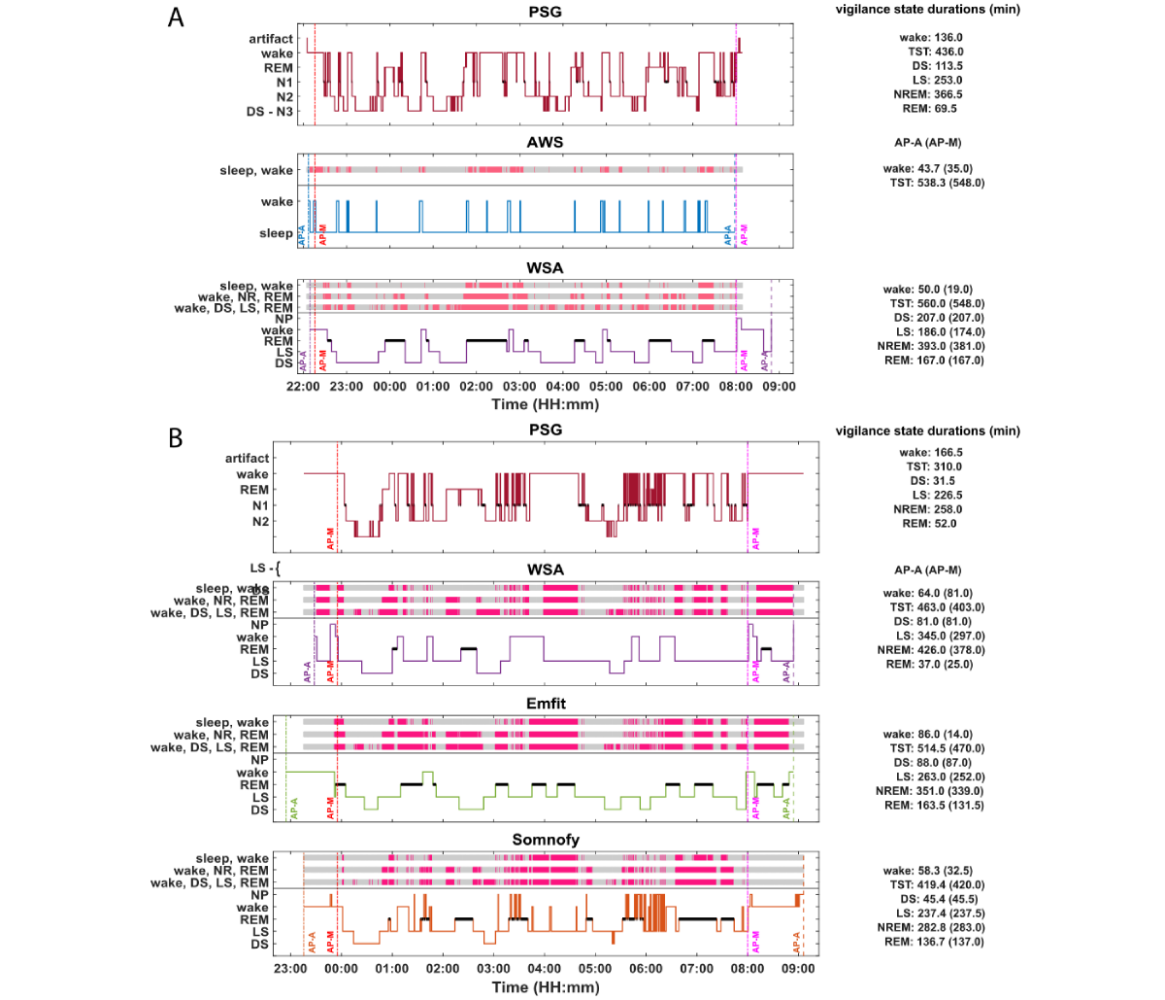
Examples of 5-stage polysomnography (PSG) hypnograms, Actiwatch Spectrum (AWS) hypnograms, and the contactless device hypnograms. (A) Example of cohort 1 (AWS and Withings sleep analyzer [WSA]). (B) Example of cohort 2 (WSA, Emfit-QS [Emfit], and Somnofy). Data are plotted for the analysis period–automatic (AP-A) or analysis period–manual (AP-M), whichever is longer. The analysis periods are marked with vertical lines. Vertical lines (red and magenta) at the beginning and end of the night indicate lights off and lights on, respectively. DS: deep sleep; LS: light sleep; N1: stage N1 of non–rapid eye movement sleep; N2: stage N2 of non–rapid eye movement sleep; N3: stage N3 of non–rapid eye movement sleep; NREM: non–rapid eye movement; REM: rapid eye movement; TST: total sleep time.

Figure 1 shows example hypnograms for 2 individuals with concurrent PSG, AWS, and CST recordings, including their EBE concordance with the PSG as well as all-night sleep summary estimates (on the right). The time series of the sleep stages is displayed for the duration of the AP as determined automatically by the device (AP-A). The numbers on the right side of the panel summarize the sleep stage duration. Values within parentheses represent the AP-A (device-determined AP), whereas the values outside parentheses are based on the AP-M (AP set from lights off to lights on). REM epochs are depicted by thick black lines. The mismatches or misclassifications between PSG and the devices are depicted above the device hypnograms for different “resolutions” (2 stages [sleep and wake], 3 stages [NREM, REM and wake], and 4 stages [DS, LS, REM, and wake]). The gray and red bars indicate the match and mismatch epochs, respectively, between the devices and PSG. All the devices have an extra label that depicts no presence or artifacts.

In these examples, the AP determined by the device does not match the PSG light off period, and sometimes the device (eg, Figure 1B, WSA) identifies sleep before lights off (Figures 1A and 1B). In these examples, the AP determined by Somnofy was closest to the PSG AP, followed by WSA and Emfit. Figures 1A and 1B illustrate that SEFF was rather low in these participants and that AWS and CSTs detected far fewer awakenings than PSG.

The example EBE concordance of the device sleep prediction compared with PSG at 3 distinct levels of sleep stage prediction (2 classes: sleep and wake; 3 classes: NREM, REM, and wake; and 4 classes: LS, DS, REM, and wake) is depicted above the hypnograms in Figure 1. From this visualization, we observe that the PSG-device EBE concordance improves with a reduction in the number of classes considered.

### AP Differences

First, we considered the period over which these devices estimate the various sleep parameters. For the CSTs, sleep summary agreement analysis was performed using both the AP automatically determined by the device (AP-A) and the summary estimate computed over the “lights off” period (AP-M). For AWS, the following three APs were considered: (1) the “automatic” AP (AP-A), (2) the “manual” AP based on lights off periods as entered in the sleep diary (sleep diary “lights off” period [AP-M1]), and (3) the “manual” AP based on the PSG “lights off” period (AP-M2). All available data were used for the agreement analysis, except for 1 WSA recording, for which the device generated a summary over a period of >24 hours.

The difference in AP depicted in Table 2 and Figure S1 and Table S4 in Multimedia Appendix 1 shows that the APs estimated by AWS (both AP-A and AP-M1) were close to the PSG AP, with no significant differences between AWS AP-A and AP-M2. Among the CSTs, on average, AP-A was longer than the lights off period by ≈70 minutes and ≈135 minutes by WSA and Emfit, respectively, whereas Somnofy AP-A was not significantly (*P*<.05) different from the lights off period (bias ≈13 min). It should be noted that because the AP-M of CSTs were manually set to correspond to the lights off period (AP-M2 in the case of AWS), the bias was 0 for this comparison and hence not reported (see Table S5 in Multimedia Appendix 1).

Metrics of agreement between the all-night sleep summary device estimates based on AP-A (AP determined by the device) and PSG estimates based on AP-M (AP set from lights off to lights on) are listed in Table 2. The values shown are mean and 95% CI.

**Table 2:** All-night sleep or wake summary measure agreement metrics.

Sleep measure		AWS-A^a^ (n=18), mean (95% CI)	WSA-A^b^ (n=34), mean (95% CI)	Emfit-A^c^ (n=16), mean (95% CI)	Somnofy-A^d^ (n=17), mean (95% CI)
**AP^e^(min)**					
	Bias (LOA^f^ down to LOA up)	−5.91 (−85.11 to 73.28)	69.99 (−40.21 to 180.19)	134.8 (61.02 to 208.58)	13.01 (−61.82 to 87.84)
	SAD^g^	0.73 (0.24 to 1.21)	1.94 (1.6 to 2.29)	3.82 (3.31 to 4.34)	0.68 (0.18 to 1.18)
	SMAPE^h^	2 (0 to 4)	7 (5 to 8)	12 (10 to 13)	2 (0 to 3)
	ICC^i^	0.34 (0 to 0.75)	0.1 (0 to 0.55)	0.64 (0 to 0.87)	0.01 (0 to 0.64)
**TST^j^ (min)**					
	Bias (LOA down to LOA up)	−11.72 (−193.62 to 170.2)	120.51 (−48.42 to 289.45)	206.22 (49.6 to 362.84)	95.74 (−32.71 to 224.18)
	SAD	0.93 (0.44 to 1.41)	2.05 (1.7 to 2.39)	3.98 (3.46 to 4.49)	1.85 (1.35 to 2.35)
	SMAPE	9 (5 to13)	15 (12 to 18)	23 (18 to 28)	13 (10 to 17)
	ICC	0.48 (0 to 0.8)	0.21 (0 to 0.61)	—^k^	0.52 (0 to 0.83)
**SOL^l^ (min)**					
	Bias (LOA down to LOA up)	7.44 (−31.0 to 45.9)	16.91 (−8.2 to 42.02)	27.19 (−12.37 to 66.74)	−5.65 (−45.84 to 34.53)
	SAD	1.18 (0.7 to 1.67)	1.25 (0.91 to 1.59)	1.79 (1.27 to 2.30)	0.98 (0.48 to 1.48)
	SMAPE	59 (42 to 76)	43 (35 to 52)	46 (31 to 61)	41 (27 to 55)
	ICC	—	0.75 (0.49 to 0.87)	0.36 (0 to 0.78)	0.1 (0 to 0.67)
**WASO^m^ (min)**					
	Bias (LOA down to LOA up)	−0.25 (−197.54 to 197.04)	−72.65 (−198.4 to 53.11)	−52.06 (−183.06 to 78.94)	−87.95 (−205.12 to 29.22)
	SAD	1.07 (0.6 to 1.56)	1.61 (1.27 to 1.96)	1.66 (1.15 to 2.18)	1.8 (1.3 to 2.3)
	SMAPE	34 (25 to 43)	44 (35 to 52)	32 (23 to 40)	51 (37 to 65)
	ICC	0.37 (0 to 0.76)	0.42 (0 to 0.71)	—	0.52 (0 to 0.83)
**SEFF^n^ (%)**					
	Bias (LOA down to LOA up)	−1.03 (−34.99 to 32.94)	12.92 (−9.96 to 35.8)	20.37 (−4.52 to 45.27)	16.66 (−8.61 to 41.94)
	SAD	1.07 (0.58 to 1.55)	1.58 (1.23 to 1.92)	2.61 (2.09 to 3.12)	1.59 (1.09 to 2.09)
	SMAPE	10 (7 to 14)	10 (8 to 12)	14 (10 to 18)	12 (8 to 16)
	ICC	0.34 (0 to 0.75)	0.45 (0 to 0.73)	—	0.57 (0 to 0.84)

^a^AWS-A: Actiwatch Spectrum automatic analysis estimates.

^b^WSA-A: Withings sleep analyzer automatic analysis estimates.

^c^Emfit-A: Emfit-QS automatic analysis estimates.

^d^Somnofy-A: Somnofy automatic analysis estimates.

^e^AP: analysis period.

^f^LOA: limit of agreement.

^g^SAD: standardized absolute difference.

^h^SMAPE: symmetric mean absolute percentage error.

^i^ICC: intraclass correlation.

^j^TST: total sleep time

^k^Not available.

^l^SOL: sleep onset latency.

^m^WASO: wake after sleep onset.

^n^SEFF: sleep efficiency.

### All-Night Sleep Summary Measures: TST, SOL, WASO, and SEFF

All-night sleep summary measures, namely TST, SOL, WASO and SEFF, computed for the automatically determined AP are presented in Table 2 and Figure S1 and Table S2 in Multimedia Appendix 1, whereas the estimates based on AP-M are presented in Table S5 in Multimedia Appendix 1. The all-night sleep summary estimates derived from AWS were, on average, very close to the PSG estimates, and the bias was not markedly different from 0 for TST, SOL, WASO, and SEFF. By contrast, most of the all-night sleep summary measures derived from the 3 CSTs deviated considerably from the PSG estimates for both AP-A and AP-M. All CSTs consistently overestimated TST (bias>90 min) and SEFF (bias>13%) and underestimated WASO (bias>50 min). WSA and Emfit overestimated SOL (bias>16 min), whereas the bias for SOL, as determined by Somnofy, was small.

The magnitude of dispersion of the difference (SAD) for AWS sleep summary estimates was considerable but was smaller than those for the CST sleep summary estimates. SAD values of the CSTs were large (>1.0) for AP and all sleep summary estimations (TST, SOL, WASO, and SEFF) apart from the Somnofy AP and SOL estimate. For all the devices, the absolute bias in the difference between the device and PSG estimates, quantified using SMAPE, was lowest for TST and SEFF (<25%).

**Figure 2:**
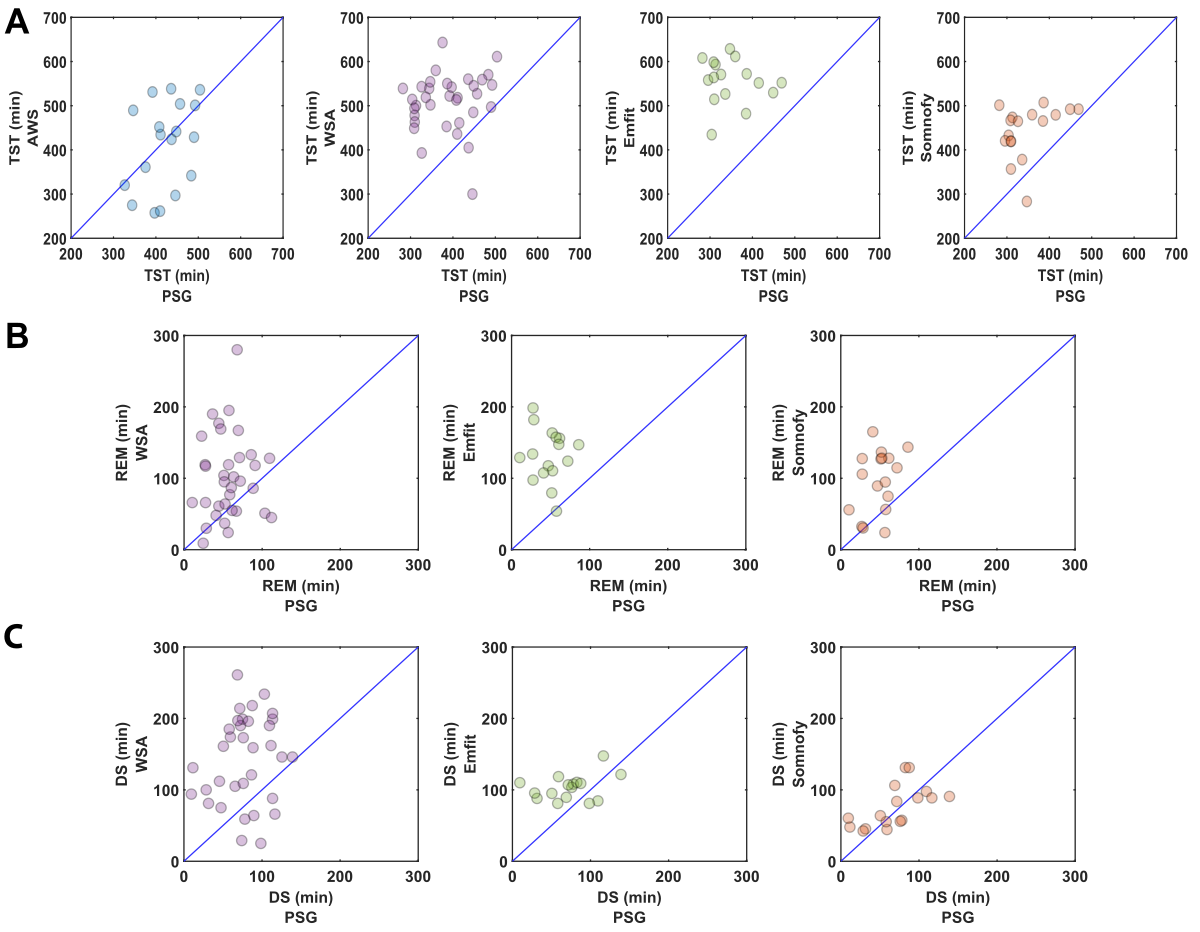
Scatter plots of all-night sleep summary measures automatically generated by the devices versus manually scored polysomnography (PSG). (A) Total sleep time (TST). (B) Rapid eye movement (REM) sleep duration. (C) Deep sleep (DS) duration. The number of participants contributing to the data of each device is as follows: 18 for Actiwatch Spectrum (AWS); 34 for Withings sleep analyzer (WSA); 16 for Emfit-QS (Emfit); and 17 for Somnofy.

The scatter plots in Figure 2A and Figure S2A in Multimedia Appendix 1 depict the overestimation, dispersion, and poor agreement of the CST TST estimates for AP-A and AP-M, respectively. They also show the considerable dispersion in AWS estimates.

For WASO and SOL, the bias was >30% for all CSTs. The results obtained from other measures of agreement such as Pearson correlation (ρ) and consistency ICC followed the Bland-Altman metrics such as bias, minimum detectable change, and dispersion measures (Tables S4 and S5 in Multimedia Appendix 1). The results of the AP-M estimates followed AP-A, except for SOL, which could be attributed to the AP that was set to the PSG lights off period.

### Sleep Stage Summary: LS, DS, REM, and NREM

The CSTs also provide a classification of sleep epochs as REM, LS, and DS. LS and DS can be combined to provide an estimate of NREM sleep. The differences in the sleep stage duration measures compared with PSG are depicted in Table 3 and Figure S1 and Tables S6 and S7 in Multimedia Appendix 1.

Metrics that depict the agreement between the sleep stage duration device estimates based on the AP-A (AP determined by the device) and PSG estimates based on AP-M (AP set from lights off to lights on) are shown in Table 3. The values shown are mean, and 95% CI.

All 3 CSTs overestimated both REM and NREM. WSA had the lowest bias (<11 min) for LS and did not show any consistent overestimation or underestimation. Somnofy (≈40 min) and Emfit (>90 min) overestimated LS, with the former having a lower bias than the latter. All CSTs had large dispersions (SAD>1.4) for REM, NREM, and LS. For DS, Somnofy had the lowest bias (<7 min) and dispersion (SAD<1) compared with the undermattress devices (bias>30 min and SAD>1.4). Among the undermattress devices, Emfit had a lower bias and dispersion.

The differences in the sleep stage duration measures were markedly, except for the LS estimates of WSA and DS estimates of Somnofy. The SMAPE was >30% for REM sleep duration across all the CSTs, and for NREM, LS, and DS, Somnofy had a lower SMAPE than the undermattress devices. The results obtained in the AP-M analysis (Table S8 in Multimedia Appendix 1) were similar to the AP-A results. Among the CSTs, only the DS duration estimates of Somnofy (both AP-A and AP-M) had satisfactory agreement (ICC>0.63).

The scatter plots in Figures 2B and 2C and Figures S2B and S2C in Multimedia Appendix 1 depict the discrepancy in the REM and DS duration estimates of the CSTs. The REM durations show overestimations and a large dispersion in the estimates across devices. For the DS duration estimate, a high level of agreement was observed for Somnofy, whereas the undermattress devices overestimated and showed large dispersion.

When the analysis was repeated for the sleep stages expressed as a percentage of TST (see Table S7 in Multimedia Appendix 1), the results were mixed. We found that WSA had the lowest percentage of bias (≈5%) and SAD for REM, followed by Somnofy and Emfit. For LS, Somnofy had the lowest bias (≈−5%) and SAD, followed by Emfit and WSA. For DS, Emfit had the lowest bias (≈−2 min), followed by Somnofy and WSA. The results were similar for the AP-M analysis (Table S9 in Multimedia Appendix 1).

Metrics that depict the agreement between the sleep stage duration device estimates based on the AP-A (AP determined by the device) and PSG estimates based on AP-M (AP set from lights off to lights on) are shown in Table 3. The values shown are mean, and 95% CI.

All 3 CSTs overestimated both REM and NREM. WSA had the lowest bias (<11 min) for LS and did not show any consistent overestimation or underestimation. Somnofy (≈40 min) and Emfit (>90 min) overestimated LS, with the former having a lower bias than the latter. All CSTs had large dispersions (SAD>1.4) for REM, NREM, and LS. For DS, Somnofy had the lowest bias (<7 min) and dispersion (SAD<1) compared with the undermattress devices (bias>30 min and SAD>1.4). Among the undermattress devices, Emfit had a lower bias and dispersion.

The differences in the sleep stage duration measures were markedly, except for the LS estimates of WSA and DS estimates of Somnofy. The SMAPE was >30% for REM sleep duration across all the CSTs, and for NREM, LS, and DS, Somnofy had a lower SMAPE than the undermattress devices. The results obtained in the AP-M analysis (Table S8 in Multimedia Appendix 1) were similar to the AP-A results. Among the CSTs, only the DS duration estimates of Somnofy (both AP-A and AP-M) had satisfactory agreement (ICC>0.63).

The scatter plots in Figures 2B and 2C and Figures S2B and S2C in Multimedia Appendix 1 depict the discrepancy in the REM and DS duration estimates of the CSTs. The REM durations show overestimations and a large dispersion in the estimates across devices. For the DS duration estimate, a high level of agreement was observed for Somnofy, whereas the undermattress devices overestimated and showed large dispersion.

When the analysis was repeated for the sleep stages expressed as a percentage of TST (see Table S7 in Multimedia Appendix 1), the results were mixed. We found that WSA had the lowest percentage of bias (≈5%) and SAD for REM, followed by Somnofy and Emfit. For LS, Somnofy had the lowest bias (≈−5%) and SAD, followed by Emfit and WSA. For DS, Emfit had the lowest bias (≈−2 min), followed by Somnofy and WSA. The results were similar for the AP-M analysis (Table S9 in Multimedia Appendix 1).

**Table 3:** Agreement metrics for sleep stage duration measures.

Sleep measure		WSA-A^a^ (n=34), mean (95% CI)	Emfit-A^b^ (n=16), mean (95% CI)	Somnofy-A^c^ (n=17), mean (95% CI)
**REM^d^ (min)**				
	Bias (LOA^e^ down to LOA up)	44.57 (−78.07 to 167.22)	84.12 (−2.01 to 170.26)	48.14 (−32.2 to 128.48)
	SAD^f^	1.27 (0.93 to 1.62)	2.93 (2.41 to 3.44)	1.59 (1.09 to 2.09)
	SMAPE^g^	33 (25 to 41)	46 (35 to 58)	34 (23 to 45)
	ICC^h^	0.07 (−0.86 to 0.54)	—^i^	0.40 (0 to 0.78)
**NREM^j^ (min)**				
	Bias (LOA down to LOA up)	75.94 (−119.47 to 271.35)	122.09 (−0.74 to 244.94)	47.59 (−68.28 to 163.45)
	SAD	1.82 (1.48 to 2.17)	2.85 (2.33 to 3.36)	1.39 (0.89 to 1.89)
	SMAPE	14 (11 to 17)	17 (12 to 22)	10 (7 to 13)
	ICC	—	—	0.33 (0 to 0.76)
**Light sleep (min)**				
	Bias (LOA down to LOA up)	10.54 (−200.13 to 221.22)	91.78 (−22.06 to 205.63)	40.85 (−76.59 to 158.29)
	SAD	1.43 (1.08 to 1.77)	2.3 (1.8 to 2.82)	1.4 (0.90 to 1.9)
	SMAPE	17 (12 to 21)	18 (13 to 23)	12 (8 to 17)
	ICC	—	0.18 (0 to 0.71)	0.12 (0 to 0.68)
**Deep sleep (min)**				
	Bias (LOA down to LOA up)	65.4 (−60.25 to 191.04)	30.31 (−33.52 to 94.15)	6.73 (−51.02 to 64.49)
	SAD	1.66 (1.31 to 2)	1.45 (0.93 to 1.96)	0.78 (0.29 to 1.28)
	SMAPE	37 (30 to 44)	25 (14 to 36)	20 (11 to 30)
	ICC	0.24 (0 to 0.62)	0.43 (0 to 0.8)	0.74 (0.3 to 0.91)

^a^WSA-A: Withings sleep analyzer automatic analysis estimates.

^b^Emfit-A: Emfit-QS automatic analysis estimates.

^c^Somnofy-A: Somnofy automatic analysis estimates.

^d^REM: rapid eye movement.

^e^SAD: standardized absolute difference.

^f^LOA: limit of agreement.

^g^SMAPE: symmetric mean absolute percentage error.

^h^ICC: intraclass correlation.

^i^Not available.

^j^NREM: non–rapid eye movement.

### DS and EEG SWA in NREM Sleep

**Figure 3:**
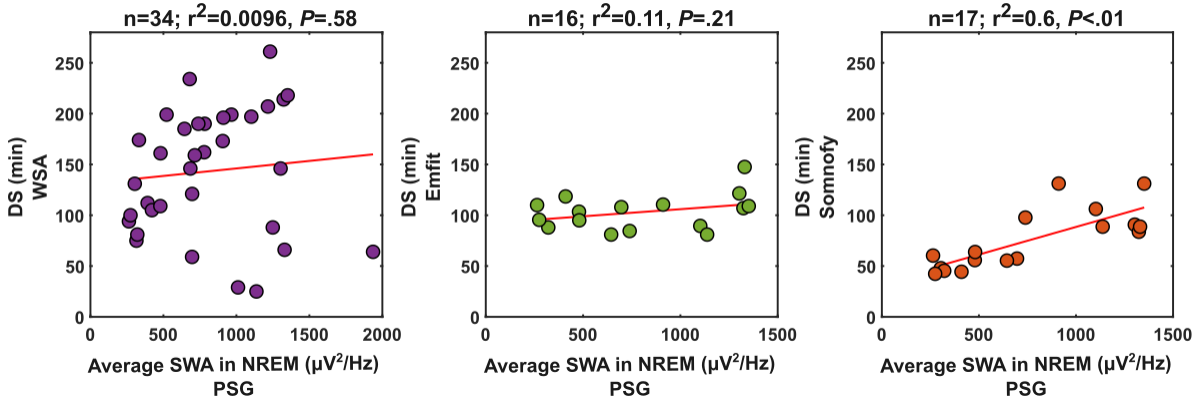
Correlation between device estimates deep sleep (DS) duration and average slow wave activity (SWA; polysomnography [PSG]) in non–rapid eye movement [NREM]. The DS duration estimates are over the analysis period–automatic of the devices. The average SWA represents the average power in the 0.75-to-4.5-Hz band in NREM epochs. r2 is the coefficient of determination of the linear model, and P is the significance level. Emfit: Emfit-QS; WSA: Withings sleep analyzer.

Visual scoring of DS is based on an amplitude and incidence criterion for slow waves such that a 30-second epoch is scored as N3 when ≥6 seconds of this epoch consists of slow waves with an amplitude equal to or greater than 75 µV [18]. Slow waves also occur in N2, and the amplitude of the slow waves declines with aging [35]. It has been repeatedly argued that DS should be quantified in a less arbitrary manner [36]. The most commonly used measure is SWA, defined as EEG power density in the range of 0.75 to 4.5 Hz, in NREM sleep. Therefore, we investigated whether DS as detected by the CSTs was associated with SWA. Somnofy DS duration was significantly correlated (*r*^2^=0.6; *P*<.01) with the average SWA detected via PSG, whereas for the undermattress devices, this correlation was not significant (WSA: *r*^2^=0.0096, *P*=.58; Emfit: *r*^2^=0.11, *P*=.21; Figure 3).

### EBE Concordance

**Figure 4:**
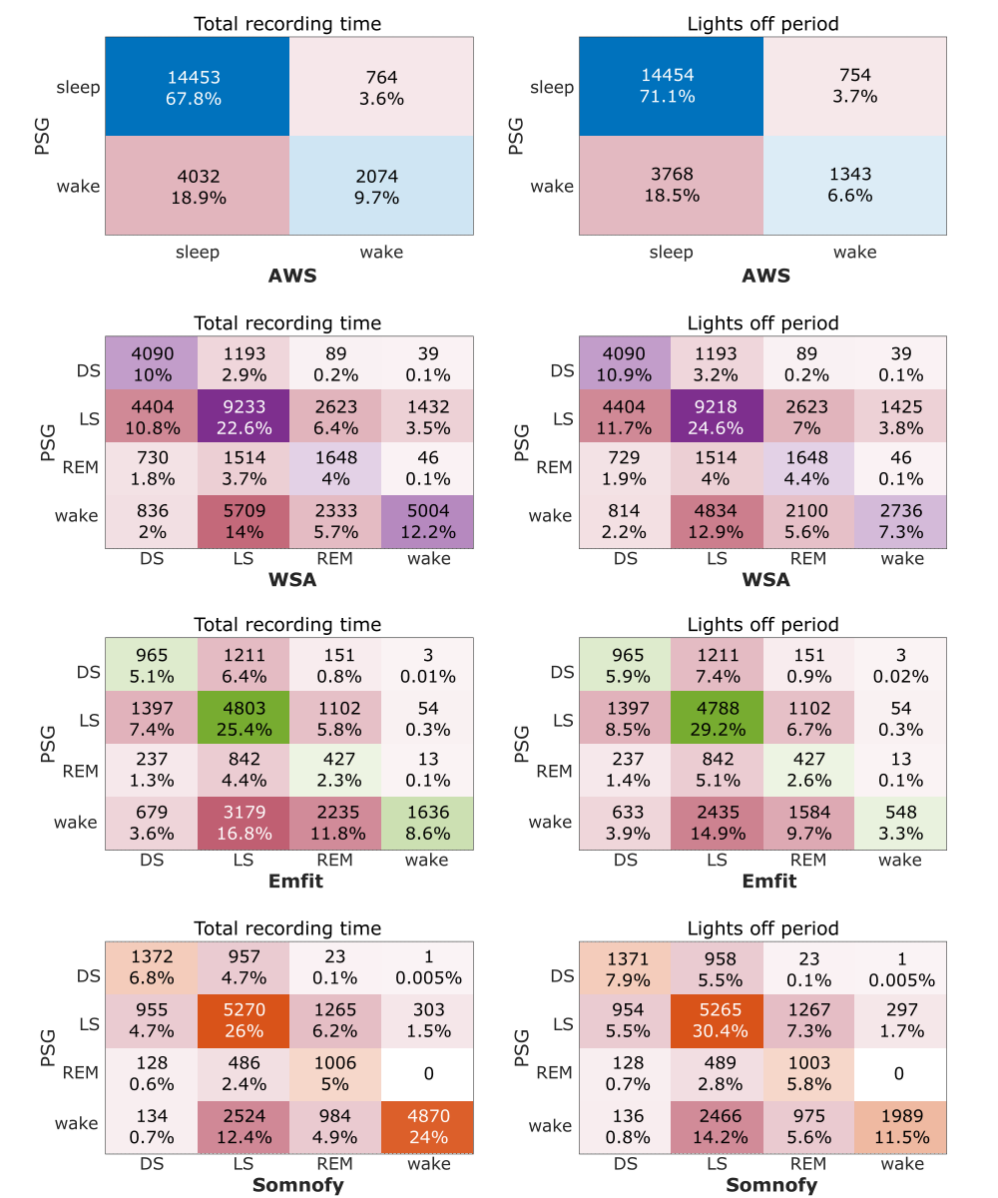
Pooled confusion matrices. The pooled confusion matrices are derived by summing participant-wise epoch-by-epoch concordance confusion matrices. The panels on the left indicate the matrices computed over the total recording time, and the panels on the right indicate the lights off period. Total number of epochs for each device for the total recording time is as follows: 21,323 for Actiwatch Spectrum (AWS); 40,923 for Withings sleep analyzer (WSA); 18,809 for Emfit-QS (Emfit); and 20,278 for Somnofy. Total number of epochs for each device for the lights off period is as follows: 20,319 for AWS; 37,502 for WSA; 16,322 for Emfit; and 17,322 for Somnofy. The number of participants contributing to the data of each device is as follows: 18 for AWS; 35 for WSA; 16 for Emfit; and 17 for Somnofy.

The CSTs generate a 4-stage sleep time series with REM, LS, DS, and wake, whereas AWS generates a 2-stage sleep-wake time series. The pooled confusion matrices for each device are shown in Figure 4. The EBE concordance between PSG and the compared devices at 3 distinct levels of sleep stage prediction resolution computed over the TRT of PSG is depicted in Table 4 and Figure S3 in Multimedia Appendix 1. The sensitivity (sleep detection accuracy) of the undermattress devices was high (>0.9), with specificity similar to (WSA) or lower (Emfit) than that of AWS. The MCC, which, unlike accuracy and specificity, is robust against class imbalance, of AWS was comparable to those of WSA and Emfit. Somnofy outperformed the undermattress devices and AWS with a moderate MCC value (0.63, 0.57-0.69). With respect to individual sleep stage prediction concordance, Somnofy showed moderate performance across all sleep stages, whereas WSA had moderate concordance for wake and DS. Emfit had poor concordance for all sleep stages. In DS estimation, WSA (MCC: 0.47, 0.42-0.52) was marginally better than Somnofy (MCC: 0.46, 0.35-0.57). The EBE concordance analysis performed over the lights off period of PSG revealed similar results to those of the TRT analysis (see Table S8 and Figure S4 in Multimedia Appendix 1). The violin plots in Figures S3 and S4 in Multimedia Appendix 1 are used to show the distribution over the participants, and each dot within the violin corresponds to the performance measure for a single participant. Among the CSTs, only the NREM EBE concordance of Somnofy for TRT had satisfactory agreement (MCC: >0.48).

The values shown in Table 4 are mean, SD, and 95% CI. Here, NREM sleep denotes epochs with either DS or LS, and sleep or wake denotes the binary sleep stage prediction performance. The metrics were computed for the TRT (from the start to the end of PSG recording) of PSG. The number of participants contributing to each device was as follows: 18 for AWS; 35 for WSA; 16 for Emfit; and 17 for Somnofy.

We further explored the EBE concordance between the CSTs and PSG using an alternate assumption of LS being equivalent to N1 and DS being equivalent to both N2 and N3, and the results are provided in Figure S5 and Table S11 in Multimedia Appendix 1. We found that the accuracy (measured through MCC) of LS detection was significantly reduced across the 3 devices compared with the original ground-truth label (LS=N1 or N2 and DS=N3), disproving the alternate assumption. This also reaffirms the ground truth that LS predicted by the CSTs is equivalent to both N1 and N2, and DS is equivalent to N3.

**Table 4:** Epoch-by-epoch agreement.

Sleep stage		Sensitivity, mean (SD; 95% CI)	Specificity, mean (SD; 95% CI)	Accuracy, mean (SD; 95% CI)	MCC^a^, mean (SD; 95% CI)	*F*_1_-score, mean (SD; 95% CI)
**Sleep or wake**						
	AWS^b^	0.95 (0.02; 0.94-0.96)	0.34 (0.12; 0.28-0.4)	0.78 (0.06; 0.74-0.81)	0.37 (0.11; 0.32-0.43)	0.85 (0.05; 0.83-0.88)
	WSA^c^	0.95 (0.09; 0.92-0.98)	0.37 (0.16; 0.31-0.42)	0.75 (0.09; 0.71-0.78)	0.41 (0.15; 0.36-0.46)	0.83 (0.07; 0.8-0.85)
	Emfit^d^	0.99 (0.03; 0.97-1)	0.22 (0.14; 0.15-0.3)	0.67 (0.11; 0.61-0.73)	0.35 (0.16; 0.26-0.43)	0.78 (0.08; 0.73-0.82)
	Somnofy	0.97 (0.06; 0.94-1)	0.58 (0.17; 0.5-0.67)	0.81 (0.08; 0.76-0.85)	0.63 (0.12; 0.57-0.69)	0.85 (0.07; 0.82-0.88)
**REM^e^**						
	WSA	0.4 (0.22; 0.32-0.48)	0.86 (0.1; 0.83-0.89)	0.82 (0.09; 0.79-0.85)	0.24 (0.16; 0.18-0.3)	0.32 (0.16; 0.27-0.38)
	Emfit	0.25 (0.19; 0.15-0.35)	0.8 (0.06; 0.77-0.83)	0.76 (0.06; 0.73-0.79)	0.12 (0.08; 0.07-0.16)	0.18 (0.11; 0.13-0.24)
	Somnofy	0.62 (0.25; 0.49-0.74)	0.88 (0.08; 0.84-0.92)	0.86 (0.08; 0.82-0.9)	0.39 (0.18; 0.3-0.49)	0.42 (0.18; 0.32-0.51)
**NREM^f^**						
	WSA	0.83 (0.12; 0.79-0.87)	0.52 (0.13; 0.47-0.56)	0.68 (0.07; 0.66-0.71)	0.38 (0.13; 0.33-0.42)	0.74 (0.06; 0.72-0.76)
	Emfit	0.83 (0.11; 0.78-0.89)	0.44 (0.13; 0.37-0.51)	0.64 (0.11; 0.58-0.7)	0.35 (0.14; 0.27-0.42)	0.7 (0.1; 0.65-0.76)
	Somnofy	0.84 (0.07; 0.81-0.88)	0.69 (0.12; 0.63-0.75)	0.76 (0.07; 0.72-0.8)	0.53 (0.13; 0.47-0.6)	0.77 (0.08; 0.73-0.81)
**Light sleep**						
	WSA	0.54 (0.13; 0.49-0.58)	0.65 (0.13; 0.61-0.7)	0.59 (0.06; 0.57-0.61)	0.2 (0.11; 0.16-0.24)	0.52 (0.08; 0.49-0.55)
	Emfit	0.63 (0.09; 0.58-0.67)	0.54 (0.1; 0.48-0.59)	0.57 (0.08; 0.53-0.61)	0.17 (0.14; 0.1-0.24)	0.53 (0.1; 0.47-0.58)
	Somnofy	0.67 (0.1; 0.62-0.72)	0.69 (0.09; 0.64-0.73)	0.68 (0.06; 0.65-0.71)	0.35 (0.14; 0.28-0.42)	0.61 (0.11; 0.55-0.66)
**Deep sleep**						
	WSA	0.79 (0.23; 0.71-0.87)	0.83 (0.09; 0.8-0.86)	0.82 (0.06; 0.8-0.84)	0.47 (0.15; 0.42-0.52)	0.51 (0.16; 0.45-0.56)
	Emfit	0.39 (0.24; 0.26-0.52)	0.85 (0.03; 0.84-0.87)	0.79 (0.05; 0.77-0.82)	0.21 (0.16; 0.13-0.29)	0.28 (0.17; 0.19-0.37)
	Somnofy	0.54 (0.21; 0.43-0.65)	0.93 (0.04; 0.91-0.95)	0.89 (0.04; 0.87-0.91)	0.46 (0.21; 0.35-0.57)	0.51 (0.21; 0.4-0.62)

^a^MCC: Matthew correlation coefficient.

^b^AWS: Actiwatch Spectrum.

^c^WSA: Withings sleep analyzer.

^d^Emfit: Emfit-QS.

^e^REM: rapid eye movement.

^f^NREM: non–rapid eye movement.

### Summarizing the Sleep Summary Measures and EBE Agreement

**Figure 5:**
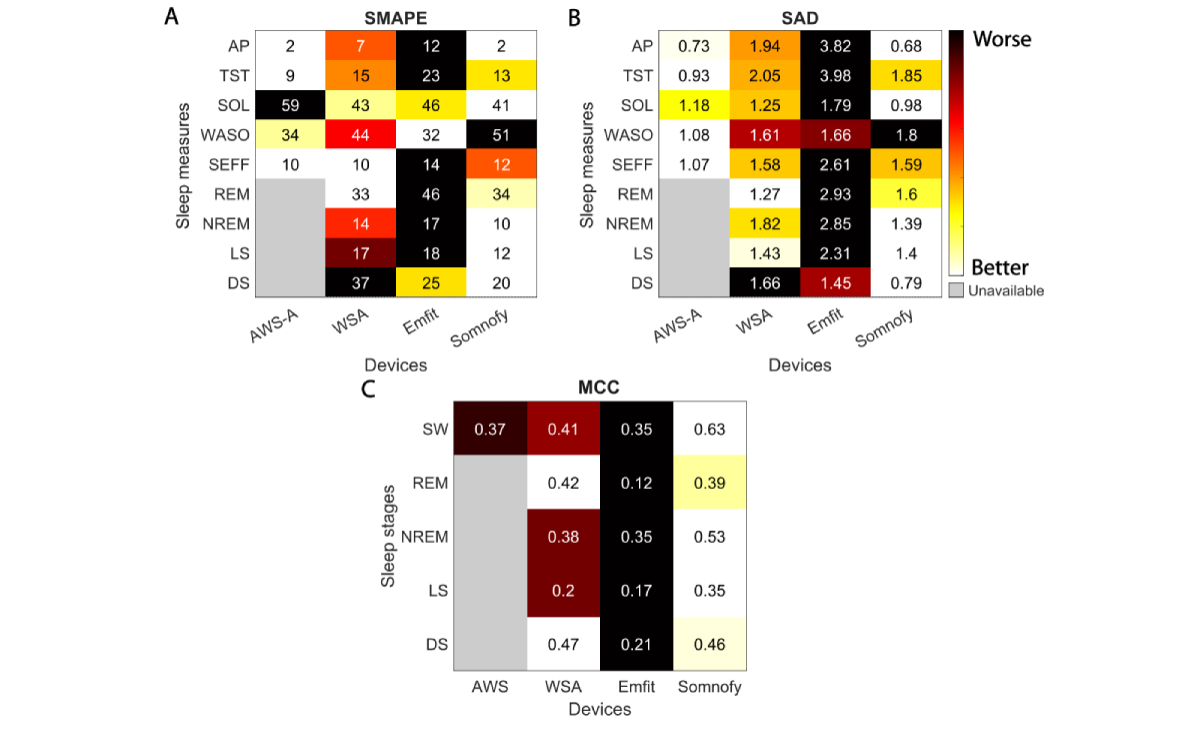
Agreement matrices depicting the sleep summary and epoch-by-epoch concordance. (A) Symmetric mean absolute percentage error (SMAPE). (B) Standardized absolute difference (SAD). The sleep summary measures are computed over the analysis period (AP)-automatic. (C) Matthew correlation coefficient (MCC, computed over the total recording time of polysomnography). The number of participants contributing to the data of each device is as follows: 18 for Actiwatch Spectrum (AWS); 34 for Withings sleep analyzer (WSA; 35 for MCC); 16 for Emfit-QS (Emfit); and 17 for Somnofy. The color code of all the agreement matrices is scaled across each row. AWS-A: Actiwatch Spectrum automatic analysis estimates; DS: deep sleep; LS: light sleep; NREM: non–rapid eye movement; REM: rapid eye movement; SEFF: sleep efficiency; SOL: sleep onset latency; SW: slow wave; TST: total sleep time; WASO: wake after sleep onset.

To provide an effective way to visualize the differences between the evaluated devices, we created heatmaps of SMAPE, SAD, and MCC (Figure 5 and Figure S6 in Multimedia Appendix 1). From these heatmaps, it appears that Somnofy is the best-performing CST, and Emfit is the worst-performing CST. We also noticed that AWS consistently outperformed the CSTs in the all-night sleep summary measure estimation, except for SOL, which was best estimated by Somnofy. We also ranked the devices using SMAPE and SAD for the all-night sleep measures and sleep stage duration estimates and MCC for EBE concordance (Tables S12 and S13 in Multimedia Appendix 1).

## Discussion

### Principal Findings

#### Overview

A comparison of 3 consumer CSTs against PSG and actigraphy in older people revealed that for all-night sleep summary measures of binary classification (sleep vs wake), CSTs did not perform as well as actigraphy. This is in line with our evaluation of CSTs against sleep diary–assisted actigraphy in an at-home setting [22]. In sleep stage classification (DS, LS, and REM), the bedside radar (Somnofy) outperformed the undermattress devices (WSA and Emfit) and had satisfactory agreement with PSG for DS duration estimate. For LS and REM estimates, the agreement was unsatisfactory for all the devices. The data were acquired from older adults with a variety of health conditions, including sleep apnea, during their first night in a sleep laboratory, and the average SEFF was only 71%. The protocol also resulted in a large interindividual variation in polysomnographic sleep-wake parameters, which contributes to the relevance of this evaluation for the intended real-world implementation of these “digital health” devices. The data imply that these contactless sleep-tracking devices provide some useful information on sleep in older people. Indeed, we have previously evaluated the CSTs against sleep diary–assisted actigraphy and have shown that the CSTs provide reliable estimates of bed occupancy in older people living at home [22]. Given their multimodal capabilities, improvements to their sleep detection algorithms to generalize results across populations could potentially lead to reliable sleep measures on par with PSG in the near future [8,37].

#### AP Differences

One of the characteristics of the quantification of sleep by contactless devices in real-world implementation is that the period over which the analysis is performed is automatically determined by the device. This contrasts with the standard polysomnographic assessments in the sleep laboratory, in which the AP is manually determined and usually set to the period from lights off to lights on. The automatically determined APs (AP-A) of AWS and Somnofy were close to the PSG lights off period (AP-M), whereas for the other devices, the AP was often very different from the PSG-based AP. These differences in performance may be related to whether the devices measure ambient light. Whereas the Somnofy bedside radar uses both changes in ambient light and bed presence information to determine the AP, the undermattress devices (WSA and Emfit) use only bed occupancy information.

Inaccurate estimation of the AP is a contributor to the relatively poor performance of WSA and Emfit with respect to the estimation of the latency to sleep onset, as these estimates improved significantly when the AP was set to the light off period. Other sleep parameters are less affected by the AP. Nevertheless, our analyses suggest that ambient light information may be useful in improving SOL estimations in CSTs and emphasize that the first target for performance improvement of these technologies is the AP.

#### Sleep Summary

The CSTs overestimate TST and SEFF and hence underestimate WASO for all AP settings. Compared with PSG, the performance of the CSTs in estimating sleep stage duration was poor, with SMAPE ranging from 10% to 46% across all devices and sleep stages. The CSTs performed less well than the wearable actigraphy device (AWS) even when the AWS AP was automatically determined. The estimation of REM sleep was particularly poor across all the devices. The DS duration estimates of Somnofy were closer to the PSG estimates, whereas the undermattress devices (WSA and Emfit) performed poorly.

#### DS and EEG SWA in NREM Sleep

The observation that the undermattress devices’ estimates of DS duration did not correlate with SWA but DS duration as detected by the Somnofy bedside radar did correlate with SWA is somewhat puzzling because all the devices use contactless ballistographic signals. Nevertheless, the superior performance of Somnofy in assessing this neurophysiological characteristic of sleep is of interest because SWA has often been proclaimed to be of particular importance for the recovery value of sleep [35].

#### EBE Concordance

The CSTs offered EBE sleep stage predictions compared with the simple sleep-wake prediction time series available in AWS. The EBE concordance of the CSTs with PSG varied across sleep stages. Overall, Somnofy had the best performance across all sleep stage predictions and satisfactory EBE concordance for NREM compared with PSG. Among the undermattress devices, WSA had a better performance than Emfit, which performed worse than AWS, even in sleep or wake discrimination [38].

### Prior Works

Overall, the all-night sleep summary and REM sleep stage duration estimation results of the CSTs in our study were in line with the observations reported by others [16,38-41]. The similar EBE sleep or wake concordance of the undermattress devices with AWS is in line with the results reported by Chinoy et al [38] for contactless devices.

#### WSA

To the best of our knowledge, there are no performance evaluation studies comparing WSA with PSG for objective sleep estimation in older adults. In a recent evaluation study by Edouard et al [24] (n=118; age: mean 49.3, SD 12.1 y), WSA overestimated TST and underestimated WASO compared with PSG, which is in line with the results obtained in our study. A notable difference is that the evaluation conducted by Edouard et al [24] was limited to TST, SEFF, and WASO estimates of WSA.

#### Somnofy

The performance of Somnofy in the estimation of TST, SEFF, and WASO in our cohorts of older people is poorer than that in a study of 71 nights by Toften et al [15] in participants without sleep disorders, whereas the DS duration estimate is similar between the 2 studies. The EBE concordance of Somnofy agrees with the overall sensitivity (0.97) estimate reported by Toften et al [15], whereas the specificity is lower in our cohort (specificity in Toften et al [15]: 0.72; specificity in our study: 0.34).

#### Emfit

The results of the Emfit evaluation in our study were congruent with those in a study by Kholghi et al [16], in which the TST was overestimated, WASO underestimated, and all sleep stage duration estimates were poor. A notable difference is that although the EBE sleep or wake discrimination sensitivity is similar (0.99), the specificity of Emfit in our study is low (0.22) but higher than that reported by Kholghi et al [16] (0.10).

It should be noted that Toften et al [15] and Kholghi et al [16] evaluated the respective devices in younger (age: mean 28.9, SD 9.7 y) and middle-aged (age: mean 53.7, SD 16.5 y) populations, respectively.

### Limitations

The primary limitations of this study are the small sample size (<20 for AWS and Emfit) and the fact that not all devices were concurrently implemented in all participants. Given the variety of confounding factors in our cohort, including age, comorbidities, and sleep disorders, the small sample size reduced the statistical power of the performance measures used, with larger error margins in the estimates and increased sensitivity to outliers. Another limitation of the study is that, owing to the proprietary nature of the devices, the data synchronization process was based on clock times and the best alignment of the device and PSG activity or movement data (for Somnofy and Emfit) and hypnograms, which is not an ideal approach. However, because all the devices were synchronized to a common network and epochs were of 30-second and 1-minute intervals, we did not find any significant synchronization issues in the study data. The final limitation of this study is the lack of transparency in the algorithms used by the different CSTs for sleep prediction and summary generation. The limited information available on the data processing pipelines involved and training set used hinders the interpretability of the evaluation results.

### Conclusions

Our inclusion or exclusion criteria were chosen such that even though the participants were in a stable health condition, several of them had comorbidities that are common in older adults [42,43]. This, together with the first night effect, resulted in mildly disturbed sleep. Our chosen population and extended period in bed contribute to the relevance of this evaluation study for assessing sleep in the real world and target populations such as people living with dementia [42]. Some of the accidental medical findings in this study, such as a case of arrhythmia (Figure S7 in Multimedia Appendix 1), provided further insights into the performance of these devices. Because the algorithms used by the CSTs for sleep staging rely on the contactless ballistographic signal, which is primarily composed of activity, breathing, and heart rate, any condition that affects cardiopulmonary function can potentially affect the performance of the algorithms. These findings point to some of the limitations of the CSTs for real-world deployment.

The study revealed that the standard actigraphy device (AWS) provides fairly accurate estimates of all-night sleep summary measures compared with PSG, but the interparticipant measurement errors were still large. From the ranking created using the various performance measures, among the CSTs, it may be concluded that overall, Somnofy outperforms the undermattress sensors. However, how useful a device depends not only on its performance but also on the use case, costs, scalability, acceptability, etc. For example, for some use cases, an estimate of the approximate TST may be sufficient, whereas for other use cases, a good EBE concordance may be important.

Overall, it can be concluded that contactless sleep-tracking devices provide some useful information on sleep behavior, but their estimates of sleep stages are not very accurate. Owing to their unintrusive nature and higher user acceptability, CSTs may offer the opportunity for clinical digital phenotyping of sleep, behavior, and health in older adults at scale in their own homes. However, our assessment underscores the clear need for improvement in the performance of CSTs across all sleep estimation domains (summary and EBE sleep) and relevant populations before they can be effectively deployed in the real world.

## Appendix

Multimedia Appendix 1 Supplemental material.

## Abbreviations

AASM: American Academy of Sleep Medicine
AHI: apnea hypopnea index
AP: analysis period
AP-A: analysis period–automatic
AP-M: analysis period–manual
AP-M1: sleep diary “lights off” period
AP-M2: polysomnography “lights off” period
AWS: Actiwatch Spectrum
CST: contactless sleep technology
DS: deep sleep
EBE: epoch-by-epoch
EEG: electroencephalography
Emfit: Emfit-QS
ESS: Epworth Sleepiness Scale
F3-M2: left frontal referenced to right mastoid
F4-M1: right frontal referenced to left mastoid
ICC: intraclass correlation
LS: light sleep
MCC: Matthew correlation coefficient
N1: stage N1 of non–rapid eye movement sleep
N2: stage N2 of non–rapid eye movement sleep
N3: stage N3 of non–rapid eye movement sleep
NREM: non–rapid eye movement
PSG: polysomnography
PSQI: Pittsburgh Sleep Quality Index
REM: rapid eye movement
SAD: standardized absolute difference
SEFF: sleep efficiency
SMAPE: symmetric mean absolute percentage error
SOL: sleep onset latency
SWA: slow wave activity
TRT: total recording time
TST: total sleep time
WASO: wake after sleep onset
WSA: Withings sleep analyzer
