# Correction: Three Contactless Sleep Technologies Compared With Actigraphy and Polysomnography in a Heterogeneous Group of Older Men and Women in a Model of Mild Sleep Disturbance: Sleep Laboratory Study

**DOI:** 10.2196/54856

**Published:** 2023-12-05

**Authors:** Kiran K G Ravindran, Ciro della Monica, Giuseppe Atzori, Damion Lambert, Hana Hassanin, Victoria Revell, Derk-Jan Dijk

**Affiliations:** 1 Surrey Sleep Research Centre, School of Biosciences, Faculty of Health and Medical Sciences University of Surrey Guildford United Kingdom; 2 UK Dementia Research Institute, Care Research and Technology Centre at Imperial College, London, and the University of Surrey Guildford United Kingdom; 3 Surrey Clinical Research Facility, School of Biosciences, Faculty of Health and Medical Sciences Guildford United Kingdom; 4 National Institute for Health Research - Royal Surrey Clinical Research Facility Guildford United Kingdom

In “Three Contactless Sleep Technologies Compared With Actigraphy and Polysomnography in a Heterogeneous Group of Older Men and Women in a Model of Mild Sleep Disturbance: Sleep Laboratory Study” (JMIR Mhealth Uhealth 2023;11:e46338) the authors noted one error.

The *P* value was erroneously swapped between the reported R-squared values of two of the compared devices. This error occurs in two places, and the following corrections have been made:

In the Results section of the Abstract, the sentence

The deep sleep duration estimates of Somnofy correlated (r^2^=0.60; *P*<.01) with electroencephalography slow wave activity (0.75-4.5 Hz) derived from PSG, whereas for the undermattress devices, this correlation was not significant (WSA: r^2^=0.0096, *P*=.21; Emfit: r^2^=0.11, *P*=.58).

has been changed to

The deep sleep duration estimates of Somnofy correlated (r^2^=0.60; *P*<.01) with electroencephalography slow wave activity (0.75-4.5 Hz) derived from PSG, whereas for the undermattress devices, this correlation was not significant (WSA: r^2^=0.0096, *P*=.58; Emfit: r^2^=0.11, *P*=.21).

In the sub section “DS and EEG SWA in NREM Sleep” of Results, the sentence

Therefore, we investigated whether DS as detected by the CSTs was associated with SWA. Somnofy DS duration was significantly correlated (r^2^=0.6; *P*<.01) with the average SWA detected via PSG, whereas for the undermattress devices, this correlation was not significant (WSA: r^2^=0.0096, *P*=.21; Emfit: r^2^=0.11, *P*=.58; Figure 3).

has been revised to

Therefore, we investigated whether DS as detected by the CSTs was associated with SWA. Somnofy DS duration was significantly correlated (r^2^=0.6; *P*<.01) with the average SWA detected via PSG, whereas for the undermattress devices, this correlation was not significant (WSA: r^2^=0.0096, *P*=.58; Emfit: r^2^=0.11, *P*=.21; Figure 3).

The correction will appear in the online version of the paper on the JMIR Publications website on December 5, 2023 together with the publication of this correction notice. Because this was made after submission to PubMed, PubMed Central, and other full-text repositories, the corrected article has also been resubmitted to those repositories.

