# Supplementary material for: Three Contactless Sleep Technologies Compared With Actigraphy and Polysomnography in a Heterogeneous Group of Older Men and Women in a Model of Mild Sleep Disturbance: Sleep Laboratory Study
Source: JMIR Mhealth Uhealth. 2023 Oct 25;11:e46338. doi: 10.2196/46338 (PMC10632916; doi:10.2196/46338)
Supplement: Multimedia Appendix 1 [file mhealth_v11i1e46338_app1.doc]

## Multimedia Appendix 1

## Contactless sleep technology deployment

All three contactless sleep technologies [CST] (Withings sleep analyser (WSA), Emfit (model name: EMFIT IP-9360 [Wi-Fi])) and Somnofy (model name: Somnofy SM-100) are wired devices which are plugged into the mains. The devices automatically detect bed presence which triggers the starts and stops of recordings. The devices were connected to a secure Wi-Fi network to enable data upload to their cloud storage maintained by the respective manufacturers. The devices automatically connected to the preset Wi-Fi network and uploaded the data at the end of each session. The recorded data were downloaded using their respective application programming interface (API) or dedicated data download web portal. Withings API and the documentation can be found in: <https://developer.withings.com/api-reference/> while the Somnofy API can be obtained via request from the manufacturer (or reference #15). Emfit documentation can be found here: <https://qs.emfit.com/docs/>.

**Table S1. Contactless sleep technology data download periods**

| **Device** | | **Data download period** |
| --- | --- | --- |
| WSA | Cohort 1 | 03.02 to 12.03.20 |
|  | Cohort 2 | 30.06 to 17.11.21 |
| Emfit | Cohort 2 | 30.06 to 17.11.21 |
| Somnofy | Cohort 2 | 14.07 to 17.11.21 |

## Analysis Period – Automatic

The additional supporting information for the estimates is provided in this appendix in tables and figures S1.

## Sleep summary definitions for Analysis Period – Manual [AP-M]

The definitions used for computing the sleep summary measures from the device/PSG hypnogram in the light off period is summarised in Supplemental Table 4 below.

**Table S2. Sleep summary measure definitions**

| **Sleep measure** | **Definition** |
| --- | --- |
| Total sleep time (TST, min) | time in minutes scored as NREM or REM but excluding epochs of Artefact/ No presence and Wake within the period between lights off and lights on |
| Sleep Onset Latency (SOL, min) | time in minutes from lights off to the first epoch of NREM or REM |
| Wake after sleep onset (WASO, min) | time in minutes of epochs scored as wake from SOL until lights on |
| Sleep Efficiency (SEFF, %) | percentage of TST against TRT |
| Sleep stage duration | time in minutes scored as sleep stage (N1, N2, N3, REM, Wake, NREM, Deep sleep, Light sleep) from lights off to lights on |

## Analysis Period – Manual [AP-M]

As discussed in the main article, we estimated the sleep summary measures of all the devices (AWS, WSA, Emfit and Somnofy) from their respective sleep stage timeseries within the lights off period (Analysis period -Manual [AP-M]) using identical sleep summary definitions as applied to PSG hypnogram (given in Supplemental Table S2). The results have been discussed in the results section of the main article and additional tables are provided in tables and figures in this appendix.

**Table S3. Device all-night sleep summary characteristics**

| **Characteristics** | **AWS-A (18)** | | **WSA-A (34)** | **Emfit-A (16)** | **Somnofy-A (17)** |
| --- | --- | --- | --- | --- | --- |
| **Estimate 1 – Analysis Period – Automatic [AP-A] (Analysis period determined by device)** | | | | | |
| **AP (minutes)** | 565.83 (41.43)  [443, 609] | | 614.76 (45.72)  [461, 692] | 647.05 (45.83)  [530.53, 709.27] | 525.4 (30.76)  [475.8, 618.35] |
| **TST (minutes)** | 410.75 (98.76)  [257.52, 538.32] | | 509.15 (65.11)  [300, 643] | 555.91 (50.54)  [434.5, 628.5] | 443.06 (59.37)  [283.27, 507.13] |
| **SOL (minutes)** | 19 (15.89)  [0, 60] | | 31.03 (16.71)  [3, 80] | 46.5 (17.94)  [19.5, 85] | 13.26 (15.99)  [1, 60.85] |
| **WASO (minutes)** | 130.19 (104.13)  [18, 315.48] | | 65.5 (53.38)  [2, 256] | 91.13 (26.76)  [46.5, 139.5] | 58.14 (47.98)  [4.5, 148.57] |
| **SEFF (%)** | 72.83 (17.29)  [43.87, 91.09] | | 84.18 (9.09)  [51, 96] | 88.78 (3.41)  [82.55, 95.15] | 84.59 (12.08)  [55, 98] |
| **DS (% of TST)** | - | | 27.67 (10.81)  [4.64, 50.39] | 18.78 (4.06)  [12.88, 28.01] | 17.05 (5.65)  [9.11, 28.22] |
| **LS (% of TST)** | - | | 52.84 (15.17)  [21.6, 87.33] | 57.74 (3.22)  [51.12, 63.66] | 61.84 (9.69)  [50.53, 81.73] |
| **REM (% of TST)** | - | | 19.49 (10.3)  [3, 45.83] | 23.46 (5.61)  [10.26, 32.65] | 21.10 (8.68)  [6.72, 35.36] |
| **Wake (minutes)** | - | | 96.03 (54.98)  [20, 290] | 91.13 (26.76)  [46.5, 139.5] | 82.34 (64.92)  [10.48, 230.33] |
|  | **AWS-M1 (18)** | **AWS-M2 (18)** | **WSA-M (35)** | **Emfit-M (16)** | **Somnofy-M (17)** |
| **Estimate 2 – Analysis Period - Manual [AP-M] (Analysis set to lights off to light on)** | | | | | |
| **AP (minutes)** | 537 (55.81)  [420, 630] | 568.44 (21.84)  [521, 586] | 542.91 (36.36)  [466, 586] | 512.25 (23.54)  [466, 539.89] | 512.39 (22.79)  [466, 539.89] |
| **TST (minutes)** | 386.9 (106.04)  [208.3, 544.51] | 417.29 (105.93)  [243.76, 548] | 477.4 (54.69)  [298, 577.5] | 488 (28.74)  [435, 533.5] | 442.29 (58.75)  [284, 507] |
| **SOL (minutes)** | 19 (18.88)  [0, 59] | 11.56 (17.89)  [0, 56] | 22.29 (26.86)  [0, 141.5] | 10.97 (15.56)  [0, 49.5] | 10.71 (10.42)  [1.5, 42] |
| **WASO (minutes)** | 121.65 (87.55)  [18, 290.8] | 131.32 (111)  [18, 332.64] | 39.37 (48.52)  [0, 256] | 13.22 (14.32)  [0, 41] | 56.74 (57.34)  [9, 205] |
| **SEFF (%)** | 71.89 (17.66)  [47.73, 92.28] | 73.53 (18.79)  [41.74, 93.84] | 88.18 (9.73)  [50.85, 98.97] | 95.28 (3.61)  [87.93, 100] | 86.48 (11.75)  [53.23, 97.43] |
| **DS (% of TST)** | - | - | 29.73 (11.28)  [4.97, 50.58] | 20.41 (4.05)  [13.67, 29.68] | 17.13 (5.67)  [9.11, 28.3] |
| **LS (% of TST)** | - | - | 51.49 (15.39)  [19.57, 87.25] | 58.59 (2.98)  [53.06, 62.88] | 61.75 (9.64)  [50.53, 81.49] |
| **REM (% of TST)** | - | - | 18.78 (10.85)  [1.58, 45.97] | 21.00 (5.37)  [10.76, 33.26] | 21.12 (8.67)  [6.73, 35.36] |
| **Wake (minutes)** | - | - | 61.46 (56.6)  [3, 284] | 22.06 (15.93)  [0, 51] | 67.18 (62.96)  [13, 247] |

Summary of device all-night sleep summary characteristics: Values shown are the mean and (standard deviation) followed by the by the range [min max]. The AWS-M1 estimate denotes the sleep summary estimated over the lights off period (AP-M1) as recorded in the sleep diary and the AWS-M2 estimate denotes the sleep summary estimated over the lights off period (AP-M2) as recorded in the polysomnography. For all the CSTs the AP-M is the lights off period of the PSG.


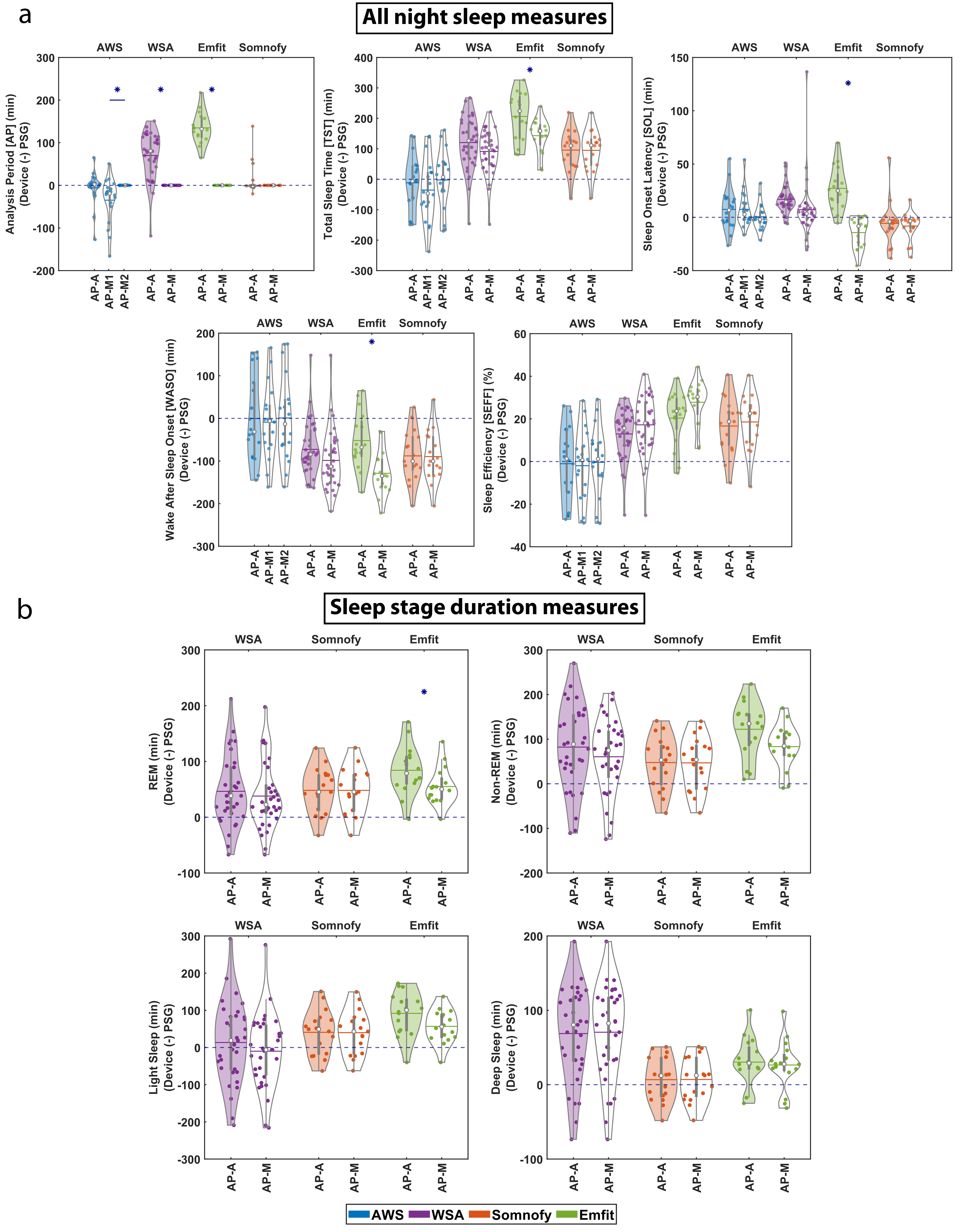


**Figure S1.** **Differences in estimations of devices against PSG (Device (-) PSG). a** all-night sleep summary measures and **b** sleep stage duration measures. The shaded violins depict the device estimates based on Analysis period – Automatic (AP-A), and the clear violins depict the Analysis period – Manual estimates (AP-M). The number of participants used in each of the devices are AWS [n=18], WSA [n=34 (AP-A)], Emfit [n=16] and Somnofy [n=17].

## Analysis Period – Automatic [AP-A]

**Table S4. All-night sleep/wake summary measure agreement metrics. [AP-A]**

| **Sleep measure** | | **AWS-A (18)** | **WSA-A (34)** | **Emfit-A (16)** | **Somnofy-A (17)** |
| --- | --- | --- | --- | --- | --- |
| **AP**  **(min)** | **Bias** | -5.91  [-85.11 73.28] | 69.99  [-40.21 180.19] | 134.8  [61.02 208.58] | 13.01  [-61.82 87.84] |
|  | **Std. Diff.** | -0.19  [-0.67 0.29] | 1.74  [1.4 2.09] | 3.82  [3.31 4.34] | 0.5  [0 0.99] |
|  | **MDC** | 79.2 | 110.2 | 73.78 | 74.83 |
|  | **Abs. Diff.** | 22.66  [5.97 39.35] | 78.07  [62.74 93.39] | 134.8  [114.74 154.86] | 17.94  [-0.57 36.45] |
|  | **SAD** | 0.73  [0.24 1.21] | 1.94  [1.6 2.29] | 3.82  [3.31 4.34] | 0.68  [0.18 1.18] |
|  | **SMAPE** | 2  [0 4] | 7  [5 8] | 12  [10 13] | 2  [0 3] |
|  | 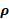 | 0.28  [-0.22 0.66] | 0.05  [-0.29 0.38] | 0.57  [0.11 0.83] | 0.01  [-0.48 0.49] |
|  | **ICC** | 0.34  [0 0.75] | 0.1  [0 0.55] | 0.64  [0 0.87] | 0.01  [0 0.64] |
|  | **t-test** | 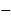 | 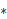 | 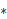 | 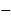 |
| **TST**  **(min)** | **Bias** | -11.72  [-193.62 170.2] | 120.51  [-48.42 289.45] | 206.22  [49.6 362.84] | 95.74  [-32.71 224.18] |
|  | **Std. Diff.** | -0.15  [-0.64 0.33] | 1.88  [1.54 2.23] | 3.98  [3.46 4.49] | 1.72  [1.22 2.22] |
|  | **MDC** | 181.9 | 168.94 | 156.62 | 128.44 |
|  | **Abs. Diff.** | 71.6  [43 100.3] | 130.99  [107.02 154.95] | 206.22  [163.64 248.8] | 103.23  [76.47 130] |
|  | **SAD** | 0.93  [0.44 1.41] | 2.05  [1.7 2.39] | 3.98  [3.46 4.49] | 1.85  [1.35 2.35] |
|  | **SMAPE** | 9  [5 13] | 15  [12 18] | 23  [18 28] | 13  [10 17] |
|  | 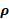 | 0.38  [-0.11 0.72] | 0.12  [-0.23 0.44] | -0.11  [-0.57 0.4] | 0.35  [-0.16 0.71] |
|  | **ICC** | 0.48  [0 0.8] | 0.21  [0 0.61] | 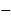 | 0.52  [0 0.83] |
|  | **t-test** | 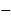 | 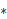 | 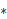 | 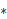 |
| **SOL**  **(min)** | **Bias** | 7.44  [-31.0 45.9] | 16.91  [-8.2 42.02] | 27.19  [-12.37 66.74] | -5.65  [-45.84 34.53] |
|  | **Std. Diff.** | 0.57  [0.08 1.05] | 1.2  [0.86 1.55] | 1.74  [1.23 2.26] | -0.39  [-0.89 0.11] |
|  | **MDC** | 38.5 | 25.11 | 39.56 | 40.18 |
|  | **Abs. Diff.** | 15.56  [8.71 22.4] | 17.56  [13.41 21.7] | 27.88  [17.67 38.08] | 14.21  [6.24 22.17] |
|  | **SAD** | 1.18  [0.7 1.67] | 1.25  [0.91 1.59] | 1.79  [1.27 2.30] | 0.98  [0.48 1.48] |
|  | **SMAPE** | 59  [42 76] | 43  [35 52] | 46  [31 61] | 41  [27 55] |
|  | 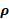 | -0.06  [-0.51 0.42] | 0.64  [0.39 0.81] | 0.22  [-0.31 0.65] | 0.05  [-0.44 0.52] |
|  | **ICC** | 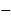 | 0.75  [0.49 0.87] | 0.36  [0 0.78] | 0.1  [0 0.67] |
|  | **t-test** | 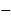 | 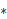 | 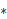 | 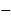 |
| **WASO**  **(min)** | **Bias** | -0.25  [-197.54 197.04] | -72.65  [-198.4 53.11] | -52.06  [-183.06 78.94] | -87.95  [-205.12 29.22] |
|  | **Std. Diff.** | -0.003  [-0.49 0.48] | -1.39  [-1.73 -1.05] | -1.21  [-1.72 -0.69] | -1.73  [-2.23 -1.23] |
|  | **MDC** | 197.3 | 125.76 | 131 | 117.17 |
|  | **Abs. Diff.** | 84.82  [59.87 109.76] | 84.18  [67.61 100.74] | 71.69  [48.60 94.77] | 91.36  [63.56 119.16] |
|  | **SAD** | 1.07  [0.6 1.56] | 1.61  [1.27 1.96] | 1.66  [1.15 2.18] | 1.8  [1.3 2.3] |
|  | **SMAPE** | 34  [25 43] | 44  [35 52] | 32  [23 40] | 51  [37 65] |
|  | 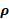 | 0.3  [-0.2 0.67] | 0.27  [-0.08 0.56] | -0.17  [-0.61 0.36] | 0.35  [-0.15 0.71] |
|  | **ICC** | 0.37  [0 0.76] | 0.42  [0 0.71] | - | 0.52  [0 0.83] |
|  | **t-test** | 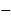 | 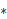 | 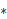 | 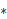 |
| **SEFF**  **(%)** | **Bias** | -1.03  [-34.99 32.94] | 12.92  [-9.96 35.8] | 20.37  [-4.52 45.27] | 16.66  [-8.61 41.94] |
|  | **Std. Diff.** | -0.08  [-0.56 0.41] | 1.34  [1 1.68] | 2.48  [1.96 2.99] | 1.47  [0.97 1.97] |
|  | **MDC** | 33.97 | 22.88 | 24.89 | 25.27 |
|  | **Abs. Diff.** | 14.24  [9.61 18.87] | 15.21  [12.3 18.13] | 21.43  [15.74 27.12] | 18.06  [12.55 23.57] |
|  | **SAD** | 1.07  [0.58 1.55] | 1.58  [1.23 1.92] | 2.61  [2.09 3.12] | 1.59  [1.09 2.09] |
|  | **SMAPE** | 10  [7 14] | 10  [8 12] | 14  [10 18] | 12  [8 16] |
|  | 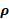 | 0.25  [-0.24 0.64] | 0.29  [-0.05 0.57] | -0.22  [-0.64 0.31] | 0.39  [-0.11 0.74] |
|  | **ICC** | 0.34  [0 0.75] | 0.45  [0 0.73] | - | 0.57  [0 0.84] |
|  | **t-test** | 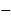 | 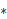 | 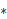 | 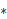 |

Metrics of agreement between the all-night sleep summary device estimates based on Analysis period – Automatic (AP-A, analysis period determined by the device) and PSG estimates (AP-M, analysis period set from lights off to lights on). The values shown are the mean followed by the [95% confidence interval]. For Bias, the mean value is followed by the Limits of agreement (LoA) [LoA-Down, LoA-Up]. The metrics include Bias – difference in measurement between the Device and PSG (Device – PSG); Standardized difference (Std. Diff.) – Cohen’s d (effect size depicting the magnitude of differences); Minimum detectable change (MDC) – smallest detectable change independent of measurement error (half of Bland Altman agreement width); Absolute difference – difference in absolute measurement value between the PSG and Device; Standardized absolute difference (SAD) – directionless version of Cohen’s d; Symmetric mean absolute percentage error (SMAPE) – mean error in measurement; Pearson’s correlation (
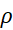
) and Consistency intraclass correlation with two-way random effects (ICC) – measures of measurement reliability; paired t-test (t-test) – significance of differences between the device and PSG (‘-’, no significant difference,
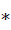
 p < 0.05). The number of participants contributing to each of the devices is represented adjacent to the device name within ‘()’. All the values are rounded to two decimal places.

## Analysis Period – Manual [AP-M]

**Table S5. Sleep/wake summary measure agreement metrics.**

| **Sleep measure** | | **AWS-M1 (18)** | **AWS-M2 (18)** | **WSA-M (35)** | **Emfit-M (16)** | **Somnofy-M (17)** |
| --- | --- | --- | --- | --- | --- | --- |
| **AP**  **(min)** | **Bias** | -34.75  [-140.37 70.87] | 0.03  [-0.52 0.58] | - | - | - |
|  | **Std. Diff.** | -0.86  [-1.34 -0.38] | 0  [-0.48 0.48] | - | - | - |
|  | **MDC** | 105.63 | 0.55 | - | - | - |
|  | **Abs. Diff.** | 43.62  [20.4 66.78] | 0.21  [0.11 0.3] | - | - | - |
|  | **SAD** | 1.08  [0.59 1.56] | 0.01  [-0.47 0.49] | - | - | - |
|  | **SMAPE** | 4  [2 6] | 0  [0 0] | - | - | - |
|  | 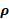 | 0.27  [-0.2 0.65] | 1  [1 1] | - | - | - |
|  | **ICC** | 0.28  [0 0.73] | 1  [1 1] | - | - | - |
|  | **t-test** | 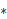 | 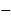 | - | - | - |
| **TST**  **(min)** | **Bias** | -35.57  [-240.84 169.7] | -2.35  [-194.18 189.48] | 91.43  [-49.28 232.14] | 138.31  [36.93 239.69] | 94.88  [-34.97 224.74] |
|  | **Std. Diff.** | -0.44  [-0.92 0.05] | -0.03  [-0.51 0.45] | 1.54  [1.2 1.87] | 3.19  [2.67 3.71] | 1.71  [1.21 2.21] |
|  | **MDC** | 205.27 | 191.83 | 140.71 | 101.38 | 129.85 |
|  | **Abs. Diff.** | 88.84  [57.59 120.1] | 76.24  [47.12 105.37] | 102.77  [84.32 121.23] | 138.31  [110.75 165.87] | 102.29  [74.91 129.68] |
|  | **SAD** | 1.09  [0.61 1.57] | 0.92  [0.44 1.4] | 1.73  [1.39 2.06] | 3.19  [2.68 3.71] | 1.85  [1.35 2.34] |
|  | **SMAPE** | 12  [7 16] | 10  [6 14] | 12  [10 0.15] | 17  [13 21] | 13  [10 17] |
|  | 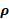 | 0.28  [-0.22 0.66] | 0.43  [-0.04 0.75] | 0.3  [-0.04 0.58] | 0.41  [-0.10 0.75] | 0.33  [-0.18 0.7] |
|  | **ICC** | 0.36  [0 0.76] | 0.51  [0 0.82] | 0.46  [0 0.73] | 0.5  [0 0.83] | 0.49  [0 0.82] |
|  | **t-test** | 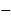 | 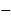 | 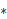 | 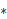 | 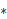 |
| **SOL**  **(min)** | **Bias** | 7.44  [-24.67, 39.57] | 0.44  [-24.2 25.09] | 7.16  [-45.57 59.88] | -8.34  [-51.91, 35.22] | -8.12  [-33.87 17.63] |
|  | **Std. Diff.** | 0.5  [0.02 0.98] | 0.03  [-0.45 0.51] | 0.35  [0.01 0.68] | -0.58  [-1.1 -0.06] | -0.69  [-1.19 -0.19] |
|  | **MDC** | 32.12 | 24.65 | 52.72 | 43.57 | 25.75 |
|  | **Abs. Diff.** | 11.72  [5.01 18.43] | 9  [4.76 13.24] | 14.33  [6.16 22.49] | 17.41  [9.06 25.75] | 10.29  [4.43 16.16] |
|  | **SAD** | 0.79  [0.3 1.27] | 0.63  [0.15 1.12] | 0.69  [0.35 1.03] | 1.21  [0.7 1.73] | 0.87  [0.37 1.37] |
|  | **SMAPE** | 45  [28 63] | 57  [38 76] | 42  [31 52] | 60  [40 81] | 33  [20 45] |
|  | 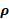 | 0.50  [0.04 0.78] | 0.71  [0.37 0.88] | 0.23  [-0.11 0.52] | -0.12  [-0.58 0.4] | 0.43  [-0.06 0.76] |
|  | **ICC** | 0.6  [0 0.85] | 0.77  [0.39 0.92] | 0.3  [-0.38 0.65] | 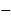 | 0.59  [0 0.85] |
|  | **t-test** | 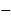 | 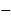 | 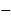 | 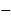 | 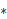 |
| **WASO**  **(min)** | **Bias** | -8.79  [-177.03 159.44] | 0.93  [-198.59 200.46] | -98.67  [-235.27 37.93] | -129.97  [-236.1 -23.84] | -89.62  [-206.76 27.52] |
|  | **Std. Diff.** | -0.13  [-0.61 0.35] | 0.01  [-0.47 0.49] | -1.99  [-2.33 -1.65] | -3.23  [-3.75 -2.72] | -1.62  [-2.12 -1.12] |
|  | **MDC** | 168.23 | 199.53 | 136.6 | 106.13 | 117.14 |
|  | **Abs. Diff.** | 65.14  [38.1 92.18] | 82.65  [54.83 110.48] | 108.61  [90.65 126.58] | 129.97  [101.11 158.82] | 94.79  [68.79 120.8] |
|  | **SAD** | 0.95  [0.47 1.43] | 1  [0.51 1.48] | 2.19  [1.86 2.53] | 3.23  [2.72 3.75] | 1.72  [1.22 2.22] |
|  | **SMAPE** | 28  [17 39] | 34  [24 44] | 65  [56 74] | 85  [77 93] | 54  [42 65] |
|  | 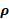 | 0.31  [-0.19 0.68] | 0.4  [-0.08 0.73] | 0.04  [-0.3 0.37] | 0.32  [-0.21 0.70] | 0.45  [-0.04 0.76] |
|  | **ICC** | 0.41  [0 0.78] | 0.45  [0 0.79] | 0.07  [0 0.53] | 0.26  [0 0.74] | 0.62  [0 0.86] |
|  | **t-test** | 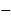 | 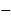 | 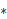 | 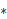 | 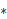 |
| **SEFF**  **(%)** | **Bias** | -1.97  [-35.26 31.33] | -0.38  [-33.76 33] | 17.2  [-9.1 43.51] | 26.87  [7.84 45.91] | 18.54  [-6.25 43.33] |
|  | **Std. Diff.** | -0.15  [-0.63 0.34] | -0.03  [-0.51 0.46] | 1.73  [1.39 2.07] | 3.25  [2.74 3.77] | 1.66  [1.16 2.15] |
|  | **MDC** | 33.29 | 33.38 | 26.31 | 19.04 | 24.79 |
|  | **Abs. Diff.** | 13.32  [8.23 18.41] | 13.29  [8.23 18.34] | 19.17  [15.62 22.71] | 26.87  [21.7 32.05] | 19.93  [14.71 25.15] |
|  | **SAD** | 0.98  [0.5 1.47] | 0.93  [0.45 1.42] | 1.93  [1.59 2.27] | 3.25  [2.74 3.77] | 1.78  [1.28 2.28] |
|  | **SMAPE** | 10  [6 14] | 10  [6 14] | 12  [10 15] | 17  [13 21] | 13  [10 17] |
|  | 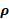 | 0.32  [-0.17 0.69] | 0.42  [-0.06 0.74] | 0.11  [-0.23 0.43] | 0.62  [0.18 0.85] | 0.4  [-0.1 0.74] |
|  | **ICC** | 0.41  [0 0.78] | 0.49  [0 0.81] | 0.2  [0 0.6] | 0.52  [0 0.83] | 0.57  [0 0.85] |
|  | **t-test** | 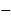 | 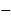 | 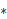 | 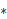 | 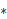 |

Metrics of agreement between the all-night sleep summary device estimates based on Device analysis period – manual estimates (AP-M, analysis period set to light off to lights on) and PSG estimates (analysis period set from lights off to lights on). The values shown are the mean followed by the 95% confidence interval. For Bias, the mean value is followed by the Limits of agreement (LoA) [LoA-Down, LoA-Up]. The metrics include Bias – difference in measurement between the Device and PSG (Device – PSG); Standardized difference (Std. Diff.) – Cohen’s d (effect size depicting the magnitude of differences); Minimum detectable change (MDC) – smallest detectable change independent of measurement error (half of Bland Altman agreement width); Absolute difference – difference in absolute measurement value between the PSG and Device; Standardized absolute difference (SAD) – directionless version of Cohen’s d; Symmetric mean absolute percentage error (SMAPE) – mean error in measurement; Pearson’s correlation (
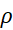
) and Consistency intraclass correlation with two-way random effects (ICC) – measures of measurement reliability; paired t-test (t-test) – significance of differences between the device and PSG (‘-’, no significant difference,
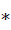
 p < 0.05). The number of participants contributing to the data of each of the devices is represented adjacent to the device name within ‘()’. All the values are rounded to two decimal places.

## Analysis Period – Automatic [AP-A]

**Table S6. Sleep stage duration summary measure agreement metrics. [AP-A]**

| **Sleep measure** | | **WSA-A (34)** | **Emfit-A (16)** | **Somnofy-A (17)** |
| --- | --- | --- | --- | --- |
| **REM**  **(min)** | **Bias** | 44.57  [-78.07 167.22] | 84.12  [-2.01 170.26] | 48.14  [-32.2 128.48] |
|  | **Std. Diff.** | 1  [0.66 1.35] | 2.91  [2.4 3.43] | 1.47  [0.97 1.97] |
|  | **MDC** | 122.64 | 86.1 | 80.3 |
|  | **Abs. Diff.** | 56.6  [38.6 74.6] | 84.56  [61.62 107.5] | 52.12  [33.7 70.34] |
|  | **SAD** | 1.27  [0.93 1.62] | 2.93  [2.41 3.44] | 1.59  [1.09 2.09] |
|  | **SMAPE** | 33  [25 41] | 46  [35 58] | 34  [23 45] |
|  | 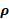 | 0.05  [-0.29 0.38] | -0.11  [-0.57 0.41] | 0.35  [-0.15 0.71] |
|  | **ICC** | 0.07  [-0.86 0.54] | 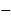 | 0.40  [0 0.78] |
|  | **t-test** | 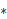 | 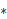 | 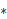 |
| **NREM**  **(min)** | **Bias** | 75.94  [-119.47 271.35] | 122.09  [-0.74 244.94] | 47.59  [-68.28 163.45] |
|  | **Std. Diff.** | 1.31  [0.96 1.65] | 2.85  [2.33 3.36] | 1.05  [0.55 1.55] |
|  | **MDC** | 195.41 | 122.84 | 115.87 |
|  | **Abs. Diff.** | 106.03  [83.15 128.91] | 122.09  [88.69 155.49] | 63.02  [41.95 84.1] |
|  | **SAD** | 1.82  [1.48 2.17] | 2.85  [2.33 3.36] | 1.39  [0.89 1.89] |
|  | **SMAPE** | 14  [11 17] | 17  [12 22] | 10  [07 13] |
|  | 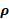 | -0.43  [-0.67 -0.1] | 0  [-0.49 0.49] | 0.2  [-0.31 0.62] |
|  | **ICC** | - | - | 0.33  [0 0.76] |
|  | **t-test** | 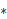 | 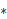 | 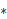 |
| **LS**  **(min)** | **Bias** | 10.54  [-200.13 221.22] | 91.78  [-22.06 205.63] | 40.85  [-76.59 158.29] |
|  | **Std. Diff.** | 0.17  [-0.17 0.52] | 2.18  [1.67 2.71] | 0.96  [0.46 1.45] |
|  | **MDC** | 210.68 | 113.8 | 117.45 |
|  | **Abs. Diff.** | 86.19  [64.08 108.3] | 96.78  [70.85 122.71] | 59.61  [39.13 80.10] |
|  | **SAD** | 1.43  [1.08 1.77] | 2.3  [1.8 2.82] | 1.4  [0.90 1.9] |
|  | **SMAPE** | 17  [12 21] | 18  [13 23] | 12  [8 17] |
|  | 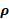 | -0.56  [-0.75 -0.27] | 0.11  [-0.41 0.57] | 0.07  [-0.43 0.53] |
|  | **ICC** | 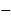 | 0.18  [0 0.71] | 0.12  [0 0.68] |
|  | **t-test** | 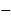 | 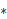 | 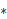 |
| **DS**  **(min)** | **Bias** | 65.4  [-60.25 191.04] | 30.31  [-33.52 94.15] | 6.73  [-51.02 64.49] |
|  | **Std. Diff.** | 1.36  [1.02 1.71] | 1.16  [0.64 1.68] | 0.21  [-0.28 0.71] |
|  | **MDC** | 125.65 | 63.8 | 57.8 |
|  | **Abs. Diff.** | 79.46  [63.81 95.1] | 37.81  [25.71 49.91] | 25.03  [16.9 33.17] |
|  | **SAD** | 1.66  [1.31 2] | 1.45  [0.93 1.96] | 0.78  [0.29 1.28] |
|  | **SMAPE** | 37  [30 44] | 25  [14 36] | 20  [11 30] |
|  | 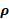 | 0.16  [-0.18 0.48] | 0.33  [-0.2 0.71] | 0.61  [0.18 0.84] |
|  | **ICC** | 0.24  [0 0.62] | 0.43  [0 0.8] | 0.74  [0.3 0.91] |
|  | **t-test** | 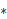 | 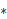 | 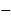 |

Metrics that depict the agreement between the sleep stage duration device estimates based on Analysis period – Automatic (analysis period determined by the device) and PSG estimates (analysis period set from lights off to lights on). The values shown are the mean (standard deviation) followed by the [95% confidence interval]. For Bias, the mean value is followed by the Limits of agreement (LoA) [LoA-Down, LoA-Up]. The metrics include Bias – difference in measurement between the Device and PSG (Device – PSG); Standardized difference (Std. Diff.) – Cohen’s d (effect size depicting the magnitude of differences); Minimum detectable change (MDC) – smallest detectable change independent of measurement error (half of Bland Altman agreement width); Absolute difference – difference in absolute measurement value between the PSG and Device; Standardized absolute difference (SAD) – directionless version of Cohen’s d; Symmetric mean absolute percentage error (SMAPE) – mean error in measurement; Pearson’s correlation (
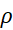
) and Consistency intraclass correlation with two-way random effects (ICC) – measures of measurement reliability; paired t-test (t-test) – significance of differences between the device and PSG (‘-’, no significant difference,
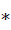
 p < 0.05). The number of participants contributing to each of the devices is represented adjacent to the device name within ‘()’. All the values are rounded to two decimal places.

## Analysis Period – Automatic [AP-A]

**Table S7. Sleep stage duration summary measure agreement metrics (% TST). [AP-A]**

| **Sleep measure** | | **WSA-A (34)** | **Emfit-A (16)** | **Somnofy-A (17)** |
| --- | --- | --- | --- | --- |
| **REM**  **(% TST)** | **Bias** | 4.93  [-19.24 29.09] | 9.97  [-5.18 25.13] | 7.33  [-9.86 24.52] |
|  | **Std. Diff.** | 0.61  [0.26 0.95] | 1.95  [1.43 2.46] | 1.07  [0.57 1.57] |
|  | **MDC** | 24.17 | 15.15 | 17.19 |
|  | **Abs. Diff.** | 9.63  [6.48 12.78] | 10.83  [7.43 14.23] | 9.41  [6.16 12.66] |
|  | **SAD** | 1.19  [0.84 1.53] | 2.11  [1.6 2.63] | 1.37  [0.87 1.87] |
|  | **SMAPE** | 28  [20 35] | 31  [20 41] | 27  [18 36] |
|  | 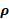 | -0.14  [-0.46 0.21] | -0.07  [-0.55 0.44] | 0.27  [-0.25 0.66] |
|  | **ICC** | 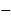 | 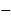 | 0.37  [0 0.77] |
|  | **t-test** | 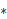 | 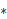 | 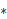 |
| **NREM**  **(% TST)** | **Bias** | -4.93  [-29.09 19.24] | -9.97  [-25.13 5.18] | -7.33  [-24.52 9.86] |
|  | **Std. Diff.** | -0.61  [-0.95 -0.26] | -1.95  [-2.46 -1.43] | -1.07  [-1.57 -0.57] |
|  | **MDC** | 24.17 | 15.15 | 17.19 |
|  | **Abs. Diff.** | 9.63  [6.48 12.78] | 10.83  [7.43 14.23] | 9.41  [6.16 12.66] |
|  | **SAD** | 1.19  [0.84 1.53] | 2.11  [1.6 2.63] | 1.37  [0.87 1.87] |
|  | **SMAPE** | 6  [4 8] | 7  [5 9] | 6  [4 8] |
|  | 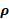 | -0.14  [-0.46 0.21] | -0.07  [-0.55 0.44] | 0.27  [-0.25 0.66] |
|  | **ICC** | 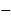 | 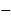 | 0.37  [0 0.77] |
|  | **t-test** | 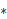 | 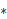 | 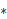 |
| **LS**  **(% TST)** | **Bias** | -12.61  [-46.75 21.52] | -8.02  [-29.32 13.28] | -4.65  [-28.12 18.82] |
|  | **Std. Diff.** | -0.99  [-1.34 -0.65] | -1  [-1.51 -0.48] | -0.45  [-0.95 0.04] |
|  | **MDC** | 34.14 | 21.3 | 23.47 |
|  | **Abs. Diff.** | 17.81  [13.67 21.94] | 9.8  [4.91 14.69] | 9.76  [5.62 13.91] |
|  | **SAD** | 1.4  [1.06 1.75] | 1.22  [0.7 1.73] | 0.95  [0.46 1.45] |
|  | **SMAPE** | 16  [12 20] | 7  [4 11] | 8  [4 11] |
|  | 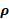 | 0.09  [-0.25 0.42] | 0.28  [-0.25 0.68] | 0.36  [-0.15 0.72] |
|  | **ICC** | 0.16  [-0.69 0.58] | 0.25  [0 0.74] | 0.53  [0 0.83] |
|  | **t-test** | 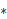 | 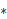 | 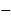 |
| **DS**  **(% TST)** | **Bias** | 7.69  [-19.95 35.33] | -1.95  [-19.24 15.33] | -2.68  [-18.39 13.03] |
|  | **Std. Diff.** | 0.82  [0.48 1.17] | -0.29  [-0.8 0.23] | -0.35  [-0.85 0.15] |
|  | **MDC** | 27.64 | 17.29 | 15.71 |
|  | **Abs. Diff.** | 13.92  [11.2 16.63] | 6.78  [3.72 9.83] | 6.36  [3.6 9.12] |
|  | **SAD** | 1.49  [1.14 1.83] | 1  [0.48 1.52] | 0.83  [0.33 1.33] |
|  | **SMAPE** | 31  [24 37] | 19  [9 30] | 20  [10 29] |
|  | 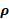 | -0.11  [-0.43 0.24] | 0.27  [-0.26 0.68] | 0.56  [0.11 0.82] |
|  | **ICC** | 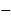 | 0.34  [-0.89 0.77] | 0.66  [0.05 0.88] |
|  | **t-test** | 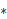 | 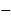 |  |

Metrics of agreement between the sleep stage duration device estimates expressed as % of total sleep time (TST) based on Device analysis period – Automatic estimates (AP-A, analysis period set to light off to lights on) and PSG estimates (analysis period set from lights off to lights on). The values shown are the mean (standard deviation) followed by the [95% confidence interval]. For Bias, the mean value is followed by the Limits of agreement (LoA) [LoA-Down, LoA-Up]. The metrics include Bias – difference in measurement between the Device and PSG (Device – PSG); Standardized difference (Std. Diff.) – Cohen’s d (effect size depicting the magnitude of differences); Minimum detectable change (MDC) – smallest detectable change independent of measurement error (half of Bland Altman agreement width); Absolute difference – difference in absolute measurement value between the PSG and Device; Standardized absolute difference (SAD) – directionless version of Cohen’s d; Symmetric mean absolute percentage error (SMAPE) – mean error in measurement; Pearson’s correlation () and Consistency intraclass correlation with two-way random effects (ICC) – measures of measurement reliability; paired t-test (t-test) – significance of differences between the device and PSG (‘-’, no significant difference, p < 0.05). The number of participants contributing to each of the devices is represented adjacent to the device name within ‘()’. All the values are rounded to two decimal places. Negative ICC values are not reported.

## Analysis Period – Manual [AP-M]

**Table S8. Sleep stage duration summary measure agreement metrics. [AP-M]**

| **Sleep measure** | | **WSA-M (35)** | **Emfit-M (16)** | **Somnofy-M (17)** |
| --- | --- | --- | --- | --- |
| **REM**  **(min)** | **Bias** | 36.4  [-81.4 154.2] | 55.06  [-11.17 121.3] | 48.12  [-32.71 128.95] |
|  | **Std. Diff.** | 0.82  [0.48 1.16] | 2.4  [1.88 2.92] | 1.47  [0.97 1.97] |
|  | **MDC** | 117.8 | 66.23 | 80.83 |
|  | **Abs. Diff.** | 50.71  [34.14 67.29] | 55.5  [37.9 73.1] | 52.06  [33.65 70.46] |
|  | **SAD** | 1.14  [0.8 1.48] | 2.42  [1.91 2.94] | 1.59  [1.09 2.09] |
|  | **SMAPE** | 33  [26 40] | 37  [26 49] | 34  [23 45] |
|  |  | 0.16  [-0.18 0.47] | -0.02  [-0.51 0.48] | 0.34  [-0.16 0.71] |
|  | **ICC** | 0.21  [0 0.6] |  | 0.40  [0 0.78] |
|  | **t-test** |  |  |  |
| **NREM**  **(min)** | **Bias** | 55.03  [-119.08 229.14] | 83.25  [-13.65 180.15] | 46.76  [-70.19 163.72] |
|  | **Std. Diff.** | 1.04  [0.7 1.38] | 2.01  [1.49 2.52] | 1.05  [0.55 1.54] |
|  | **MDC** | 174.11 | 96.9 | 116.95 |
|  | **Abs. Diff.** | 89.94  [72.16 107.73] | 85.06  [60.53 109.59] | 63.76  [43.43 84.1] |
|  | **SAD** | 1.7  [1.36 2.04] | 2.05  [1.53 2.57] | 1.43  [0.93 1.93] |
|  | **SMAPE** | 13  [10 15] | 13  [9 16] | 10  [7 13] |
|  |  | -0.38  [-0.63 -0.05] | 0.36  [-0.17 0.72] | 0.16  [-0.34 0.6] |
|  | **ICC** |  | 0.5  [0 0.83] | 0.28  [0 0.74] |
|  | **t-test** |  |  |  |
| **LS**  **(min)** | **Bias** | -12.2  [-201.24 176.84] | 56.78  [-29.41 142.97] | 39.82  [-76.34 155.98] |
|  | **Std. Diff.** | -0.22  [-0.56 0.12] | 1.48  [0.96 2] | 0.95  [0.45 1.45] |
|  | **MDC** | 189.04 | 86.19 | 116.17 |
|  | **Abs. Diff.** | 74.06  [52.86 95.26] | 61.78  [42.56 81.0] | 58.24  [37.71 78.77] |
|  | **SAD** | 1.34  [1 1.68] | 1.61  [1.09 2.13] | 1.39  [0.89 1.89] |
|  | **SMAPE** | 15  [11 19] | 13  [8 17] | 12  [8 16] |
|  |  | -0.48  [-0.7 -0.18] | 0.47  [-0.03 0.78] | 0.06  [-0.43 0.53] |
|  | **ICC** |  | 0.56  [0 0.85] | 0.11  [0 0.68] |
|  | **t-test** |  |  |  |
| **DS**  **(min)** | **Bias** | 67.23  [-58.46 192.92] | 26.47  [-38.26 91.2] | 6.94  [-50.84 64.73] |
|  | **Std. Diff.** | 1.41  [1.07 1.75] | 0.99  [0.48 1.51] | 0.22  [-0.28 0.72] |
|  | **MDC** | 125.69 | 64.73 | 57.79 |
|  | **Abs. Diff.** | 80.89  [65.41 96.36] | 36.09  [24.81 47.38] | 25.06  [16.88 33.24] |
|  | **SAD** | 1.7  [1.36 2.04] | 1.35  [0.84 1.87] | 0.79  [0.29 1.29] |
|  | **SMAPE** | 38  [31 44] | 24  [13 35] | 20  [11 30] |
|  |  | 0.15  [-0.2 0.46] | 0.33  [-0.2 0.71] | 0.61  [0.18 0.84] 125.69 |
|  | **ICC** | 0.21  [-0.56 0.6] | 0.44  [0 0.8] | 0.75  [0.3 0.91] |
|  | **t-test** |  |  |  |

Metrics of agreement between the sleep stage duration device estimates based on Device analysis period – manual estimates (AP-M, analysis period set to light off to lights on) and PSG estimates (analysis period set from lights off to lights on). The values shown are the mean (standard deviation) followed by the 95% confidence interval. The metrics include Bias – difference in measurement between the Device and PSG (Device – PSG); Standardized difference (Std. Diff.) – Cohen’s d (effect size depicting the magnitude of differences); Minimum detectable change (MDC) – smallest detectable change independent of measurement error (half of Bland Altman agreement width); Absolute difference – difference in absolute measurement value between the PSG and Device; Standardized absolute difference (SAD) – directionless version of Cohen’s d; Symmetric mean absolute percentage error (SMAPE) – mean error in measurement; Pearson’s correlation () and Consistency intraclass correlation with two-way random effects (ICC) – measures of measurement reliability; paired t-test (t-test) – significance of differences between the device and PSG (‘-’, no significant difference, p < 0.05). The number of participants contributing to each of the devices is represented adjacent to the device name within ‘()’. All the values are rounded to two decimal places.

## Analysis Period – Manual [AP-M]

**Table S9. Sleep stage duration summary measure agreement metrics (% TST). [AP-M]**

| **Sleep measure** | | **WSA-M (35)** | **Emfit-M (16)** | **Somnofy-M (17)** |
| --- | --- | --- | --- | --- |
| **REM**  **(% TST)** | **Bias** | 4.38  [-19.62 28.37] | 7.51  [-7.22 22.24] | 7.35  [-9.92 24.61] |
|  | **Std. Diff.** | 0.52  [0.18 0.86] | 1.5  [0.99 2.02] | 1.07  [0.57 1.57] |
|  | **MDC** | 23.99 | 14.73 | 17.27 |
|  | **Abs. Diff.** | 9.29  [6.2 12.38] | 8.3  [4.81 11.8] | 9.45  [6.19 12.7] |
|  | **SAD** | 1.1  [0.76 1.44] | 1.66  [1.14 2.18] | 1.38  [0.88 1.88] |
|  | **SMAPE** | 28  [20 35] | 25  [14 36] | 27  [18 36] |
|  |  | -0.02  [-0.35 0.32] | -0.06  [-0.54 0.45] | 0.26  [-0.25 0.66] |
|  | **ICC** |  |  | 0.36  [0 0.77] |
|  | **t-test** |  |  |  |
| **NREM**  **(% TST)** | **Bias** | -4.38  [-28.37 19.62] | -7.51  [-22.24 7.22] | -7.35  [-24.61 9.92] |
|  | **Std. Diff.** | -0.52  [-0.86 -0.18] | -1.5  [-2.02 -0.99] | -1.07  [-1.57 -0.57] |
|  | **MDC** | 23.99 | 14.73 | 17.27 |
|  | **Abs. Diff.** | 9.29  [6.2 12.38] | 8.3  [4.81 11.8] | 9.45  [6.19 12.7] |
|  | **SAD** | 1.1  [0.76 1.44] | 1.66  [1.14 2.18] | 1.38  [0.88 1.88] |
|  | **SMAPE** | 6  [4 8] | 5  [3 7] | 6  [4 8] |
|  |  | -0.02  [-0.35 0.32] | -0.06  [-0.54 0.45] | 0.26  [-0.25 0.66] |
|  | **ICC** |  |  | 0.36  [0 0.77] |
|  | **t-test** |  |  |  |
| **LS**  **(% TST)** | **Bias** | -14.12  [-48.19 19.94] | -7.18  [-27.07 12.72] | -4.74  [-28.06 18.58] |
|  | **Std. Diff.** | -1.11  [-1.44 -0.77] | -0.9  [-1.41 -0.38] | -0.46  [-0.96 0.03] |
|  | **MDC** | 34.07 | 19.9 | 23.32 |
|  | **Abs. Diff.** | 18.93  [14.89 22.98] | 8.66  [3.95 13.37] | 9.68  [5.5 13.85] |
|  | **SAD** | 1.48  [1.14 1.82] | 1.08  [0.57 1.6] | 0.95  [0.45 1.45] |
|  | **SMAPE** | 17  [13 21] | 6  [3 10] | 7  [4 11] |
|  |  | 0.11  [-0.23 0.43] | 0.5  [0.01 0.8] | 0.37  [-0.14 0.72] |
|  | **ICC** | 0.18  [0 0.59] | 0.4  [0 0.79] | 0.53  [0 0.83] |
|  | **t-test** |  |  |  |
| **DS**  **(% TST)** | **Bias** | 9.75  [-18.25 37.75] | -0.33  [-17.97 17.31] | -2.6  [-18.19 12.98] |
|  | **Std. Diff.** | 1.02  [0.68 1.36] | -0.05  [-0.56 0.47] | -0.34  [-0.84 0.16] |
|  | **MDC** | 28 | 17.64 | 15.58 |
|  | **Abs. Diff.** | 15.07  [12.23 17.9] | 6.35  [3.06 9.64] | 6.32  [3.59 9.04] |
|  | **SAD** | 1.57  [1.23 1.91] | 0.94  [0.42 1.45] | 0.82  [0.32 1.32] |
|  | **SMAPE** | 32  [25 38] | 18  [7 29] | 19  [10 29] |
|  |  | -0.09  [-0.41 0.26] | 0.23  [-0.3 0.65] | 0.57  [0.12 0.82] |
|  | **ICC** |  | 0.29  [0 0.75] | 0.66  [0.07 0.88] |
|  | **t-test** |  |  |  |

Metrics of agreement between the sleep stage duration device estimates expressed as % of total sleep time (TST) based on Device analysis period – manual estimates (AP-M, analysis period set to light off to lights on) and PSG estimates (analysis period set from lights off to lights on). The values shown are the mean (standard deviation) followed by the 95% confidence interval. The metrics include Bias – difference in measurement between the Device and PSG (Device – PSG); Standardized difference (Std. Diff.) – Cohen’s d (effect size depicting the magnitude of differences); Minimum detectable change (MDC) – smallest detectable change independent of measurement error (half of Bland Altman agreement width); Absolute difference – difference in absolute measurement value between the PSG and Device; Standardized absolute difference (SAD) – directionless version of Cohen’s d; Symmetric mean absolute percentage error (SMAPE) – mean error in measurement; Pearson’s correlation () and Consistency intraclass correlation with two-way random effects (ICC) – measures of measurement reliability; paired t-test (t-test) – significance of differences between the device and PSG (‘-’, no significant difference, p < 0.05). The number of participants contributing to each of the devices is represented adjacent to the device name within ‘()’. All the values are rounded to two decimal places.

## Analysis Period – Manual [AP-M]

**Figure S2.** Scatter plots of all-night sleep summary measures computed over the analysis period – manual [AP-M] by the devices vs polysomnography. a Total sleep time (TST); b Rapid eye movement (REM) sleep duration and c Deep sleep (DS) duration. The 45 degrees indicate the line of perfect agreement between the polysomnography and device estimates. The number of participants contributing to the data of each of the devices are AWS [n=18], WSA [34], Emfit [n=16] and Somnofy [n=17].

## Analysis Period – Automatic [AP-A]

**Figure S3. Epoch by epoch (EBE) concordance across devices.** The concordance is computed for the total recording period of the PSG. There are three different hypnogram resolutions depicted in the figure: a) two stages: Sleep and Wake; b) three stages: NREM, REM and Wake and c) four stages: deep sleep (DS), light sleep (LS), rapid eye movement (REM) and Wake. The number of participants used in each of the devices are AWS [n=18], WSA [n=35], Somnofy [n=17] and Emfit [n=16].

## Analysis Period – Manual [AP-M]

**Table S10. Epoch by epoch (EBE) agreement [AP-M].**

| **Sleep Stage** | | **Sensitivity** | **Specificity** | **Accuracy** | **MCC** | **F1 Score** |
| --- | --- | --- | --- | --- | --- | --- |
| Sleep/Wake | AWS | 0.95 (0.02)  [0.94, 0.96] | 0.26 (0.11)  [0.21, 0.32] | 0.78 (0.07)  [0.74, 0.81] | 0.29 (0.1)  [0.24, 0.34] | 0.86 (0.05)  [0.84, 0.89] |
|  | WSA | 0.95 (0.09)  [0.92, 0.98] | 0.26 (0.17)  [0.2, 0.32] | 0.75 (0.1)  [0.72, 0.79] | 0.3 (0.14)  [0.25, 0.35] | 0.84 (0.07)  [0.82, 0.87] |
|  | Emfit | 0.99 (0.03)  [0.97, 1] | 0.12 (0.09)  [0.07, 0.16] | 0.71 (0.11)  [0.65, 0.77] | 0.24 (0.14)  [0.17, 0.32] | 0.82 (0.08)  [0.78, 0.86] |
|  | Somnofy | 0.97 (0.06)  [0.94, 1] | 0.34 (0.23)  [0.23, 0.46] | 0.78 (0.1)  [0.73, 0.83] | 0.45 (0.14)  [0.37, 0.52] | 0.85 (0.07)  [0.82, 0.89] |
| REM | WSA | 0.4 (0.22)  [0.32, 0.48] | 0.85 (0.11)  [0.82, 0.89] | 0.81 (0.09)  [0.78, 0.84] | 0.24 (0.17)  [0.18, 0.3] | 0.33 (0.16)  [0.28, 0.39] |
|  | Emfit | 0.25 (0.19)  [0.15, 0.35] | 0.81 (0.06)  [0.77, 0.84] | 0.76 (0.06)  [0.72, 0.79] | 0.13 (0.1)  [0.08, 0.18] | 0.20 (0.12)  [0.14, 0.27] |
|  | Somnofy | 0.61 (0.25)  [0.48, 0.74] | 0.85 (0.09)  [0.81, 0.9] | 0.83 (0.09)  [0.79, 0.88] | 0.38 (0.18)  [0.29, 0.48] | 0.42 (0.18)  [0.32, 0.51] |
| NREM | WSA | 0.83 (0.12)  [0.79, 0.87] | 0.46 (0.14)  [0.41, 0.51] | 0.68 (0.07)  [0.65, 0.7] | 0.32 (0.13)  [0.28, 0.37] | 0.76 (0.06)  [0.74, 0.78] |
|  | Emfit | 0.83 (0.11)  [0.78, 0.89] | 0.37 (0.07)  [0.33, 0.41] | 0.64 (0.1)  [0.59, 0.69] | 0.27 (0.11)  [0.21, 0.33] | 0.73 (0.1)  [0.68, 0.78] |
|  | Somnofy | 0.84 (0.07)  [0.81, 0.88] | 0.57 (0.15)  [0.49, 0.65] | 0.72 (0.09)  [0.68, 0.77] | 0.42 (0.15)  [0.35, 0.5] | 0.77 (0.08)  [0.73, 0.82] |
| Light Sleep | WSA | 0.54 (0.13)  [0.49, 0.58] | 0.63 (0.15)  [0.58, 0.68] | 0.58 (0.07)  [0.55, 0.6] | 0.18 (0.1)  [0.15, 0.22] | 0.53 (0.08)  [0.5, 0.56] |
|  | Emfit | 0.63 (0.09)  [0.58, 0.67] | 0.5 (0.07)  [0.46, 0.53] | 0.56 (0.07)  [0.52, 0.59] | 0.14 (0.11)  [0.08, 0.2] | 0.55 (0.1)  [0.5, 0.61] |
|  | Somnofy | 0.67 (0.1)  [0.62, 0.72] | 0.59 (0.11)  [0.54, 0.65] | 0.63 (0.07)  [0.59, 0.67] | 0.27 (0.15)  [0.19, 0.34] | 0.61 (0.11)  [0.56, 0.67] |
| Deep Sleep | WSA | 0.79 (0.23)  [0.71, 0.87] | 0.81 (0.1)  [0.78, 0.84] | 0.8 (0.07)  [0.78, 0.83] | 0.46 (0.15)  [0.41, 0.51] | 0.51 (0.16)  [0.45, 0.56] |
|  | Emfit | 0.39 (0.24)  [0.26, 0.52] | 0.83 (0.03)  [0.82, 0.85] | 0.77 (0.05)  [0.74, 0.79] | 0.2 (0.15)  [0.12, 0.28] | 0.29 (0.17)  [0.2, 0.38] |
|  | Somnofy | 0.54 (0.21)  [0.43, 0.65] | 0.93 (0.04)  [0.9, 0.94] | 0.87 (0.04)  [0.85, 0.89] | 0.45 (0.21)  [0.34, 0.56] | 0.51 (0.21)  [0.4, 0.62] |

The values shown are the mean (standard deviation) and the [95% confidence interval]. Here non rapid eye movement (NREM) sleep denotes epochs with either deep sleep or light sleep and sleep/wake denotes the binary sleep stage discrimination performance. The metrics were computed over the lights off period of the PSG. The number of participants contributing to each of the devices are AWS [n=18], WSA [n=35], Emfit [n=16] and Somnofy [n=17]. All the values are rounded to two decimal places.

## EBE concordance between the CSTs and PSG using an alternate assumption.

**Table S11. Epoch by epoch (EBE) agreement (assumption LS=N1 and DS= N2/N3).**

| **Sleep Stage** | | **Matthew’s CC**  **(LS=N1/N2 & DS= N3)** | **Matthew’s CC**  **(LS=N1 & DS= N2/N3)** | **p (Effect size)** |
| --- | --- | --- | --- | --- |
| REM | WSA | 0.24 (0.16)  [0.18 0.3] | 0.24 (0.16)  [0.18 0.3] | - |
|  | Emfit | 0.12 (0.08)  [0.07 0.16] | 0.12 (0.08)  [0.07 0.16] | - |
|  | Somnofy | 0.39 (0.18)  [0.3 0.49] | 0.39 (0.18)  [0.3 0.49] | - |
| NREM | WSA | 0.38 (0.13)  [0.33 0.42] | 0.38 (0.13)  0.33 0.42 | - |
|  | Emfit | 0.35 (0.14)  [0.27 0.42] | 0.35 (0.14)  [0.27 0.42] | - |
|  | Somnofy | 0.53 (0.13)  [0.47 0.6] | 0.53 (0.13)  [0.47 0.6] | - |
| Light Sleep | WSA | 0.2 (0.11)  [0.16 0.24] | 0.08 (0.06)  [0.06 0.11] | **<0.001 (1.31)** |
|  | Emfit | 0.17 (0.14)  [0.1 0.24] | 0.09 (0.06)  [0.06 0.12] | **0.006 (1.07)** |
|  | Somnofy | 0.35 (0.14)  [0.28 0.42] | 0.11 (0.07)  [0.08 0.14] | **<0.001 (2.33)** |
| Deep Sleep | WSA | 0.47 (0.15)  [0.42 0.52] | 0.37 (0.12)  [0.33 0.41] | **0.002 (0.79)** |
|  | Emfit | 0.21 (0.16)  [0.13 0.29] | 0.21 (0.09)  [0.17 0.26] | 0.43 (0.29) |
|  | Somnofy | 0.46 (0.21)  [0.35 0.57] | 0.35 (0.1)  [0.3 0.41] | 0.067(0.67) |

The values shown are the mean (standard deviation) followed by the 95% confidence interval. The metrics were computed for the total recording time (TRT, start to end of polysomnography (PSG) recording) of the PSG. The number of participants contributing to each of the devices are AWS [n=18], WSA [n=35], Emfit [n=16] and Somnofy [n=17]. Significance of difference between the true label (LS=N1/N2 & DS= N3) and assumed labels (LS=N1 & DS= N2/N3) is given along with the effect size (Cohen’s D). Significant differences p<0.05 are highlighted in bold. All the values are rounded to two decimal places.

## Summarizing the Sleep Summary Measures and EBE Agreement

## Analysis Period – Automatic [AP-A]

**Table S12. Device performance rank table. [AP-A]**

| **Metric** | **AWS-A** | **WSA** | **Emfit** | **Somnofy** |
| --- | --- | --- | --- | --- |
| **Sleep summary measures** | | | | |
| AP | 2 | 3 (2) | 4 (3) | 1 (1) |
| TST | 1 | 3 (2) | 4 (3) | 2(1) |
| SOL | 3 | 2 (2) | 4 (3) | 1 (1) |
| WASO | 1 | 3(2) | 2(1) | 4(3) |
| SEFF | 1 | 2(1) | 4(3) | 3(2) |
| REM Duration | - | 1 | 3 | 2 |
| NREM Duration | - | 2 | 3 | 1 |
| Light Sleep Duration | - | 2 | 3 | 1 |
| Deep Sleep Duration | - | 3 | 2 | 1 |
| **Epoch-by-Epoch concordance** | | | | |
| Sleep/Wake | 3 | 2(2) | 4(3) | 1(1) |
| REM | - | 2 | 3 | 1 |
| NREM | - | 2 | 3 | 1 |
| Light Sleep | - | 2 | 3 | 1 |
| Deep Sleep | - | 1 | 3 | 2 |

Ranking is ordinal where every device is given a unique ranking to denote overall performance. The ranking is created using the agreement matrices shown in Figure 4. For the all-night sleep summary measure ranking, ranking is created for both symmetric mean absolute percentage error (SMAPE) and standardized absolute difference (SAD) and averaged to get the final device rank. Two devices with the same average ranking are given the same rank. The rank within ‘()’ depict the ranking among the CST subgroups. 1= best performance.

## Analysis Period – Manual [AP-M]

**Table S13. Device performance rank table. [AP-M]**

| **Metric** | **AWS-M1** | **AWS-M2** | **WSA** | **Emfit** | **Somnofy** |
| --- | --- | --- | --- | --- | --- |
| **Sleep summary measures** | | | | | |
| TST | 2 | 1 | 3 (1) | 4 (2) | 3 (1) |
| SOL | 2 | 3 | 3 (2) | 4 (3) | 1 (1) |
| WASO | 1 | 2 | 3 (2) | 2 (3) | 2 (1) |
| SEFF | 1 | 1 | 2 (1) | 3 (2) | 2 (1) |
| REM Duration | - | - | 1 | 3 | 2 |
| NREM Duration | - | - | 2 | 3 | 1 |
| Light Sleep Duration | - | - | 2 | 3 | 1 |
| Deep Sleep Duration | - | - | 3 | 2 | 1 |
| **Epoch-by-Epoch concordance** | | | | | |
| Sleep/Wake | 3 | 3 | 2 (2) | 4 (3) | 1 (1) |
| REM | - | - | 2 | 3 | 1 |
| NREM | - | - | 2 | 3 | 1 |
| Light Sleep | - | - | 2 | 3 | 1 |
| Deep Sleep | - | - | 1 | 3 | 2 |

Ranking is ordinal where every device is given a unique ranking to denote overall performance. The ranking is created using the agreement matrices. For the all night sleep summary measure ranking, the device ranking is created for both SMAPE and SAD and the final ranking is created using the average rank. Two devices with the same average ranking are given the same rank. The rank within ‘()’ depicts the ranking among the contactless device subgroup.

## Analysis Period – Manual [AP-M]

**Figure S4. Epoch by epoch (EBE) concordance across devices.** The concordance is computed for the lights off period of the PSG. There are three different hypnogram resolutions depicted in the figure: a) two stages: Sleep and Wake, b) three stages: NREM, REM and Wake and c) four stages: deep sleep (DS), light sleep (LS), rapid eye movement (REM) and Wake. Each violin depicts the distribution of estimates. The boxplot at the centre of the violin depicts the median (circle at the centre), 1st and 3rd quartiles (lower and upper ends of the box) and the whiskers depict the 1.5 × IQR (inter quartile range). The solid-coloured line in the violins depict the mean. The number of participants contributing to each of the devices are AWS [n=18], WSA [n=35], Emfit [n=16] and Somnofy [n=17].

## Pooled confusion matrices between the CSTs and PSG using an alternative assumption

**Figure S5. Pooled Confusion Matrices (assumption LS=N1 and DS= N2/N3).** The pooled confusion matrices are derived by summing participant wise epoch-by-epoch (EBE) concordance confusion matrices for the assumption of light sleep (LS) = N1 and deep sleep (DS) = N2 /N3. The panels on the left indicate the matrices computed over the total recording time (TRT) and the panel on the right indicate the lights off period. Total number of epochs for each device for the total recording time [AWS- 21323; WSA- 40923; Emfit- 18809; Somnofy- 20278] and lights off period [AWS- 20319; WSA- 37502; Emfit- 16322; Somnofy- 17322]. The number of participants contributing to the data of each of the devices are AWS [n=18], WSA [35], Emfit [n=16] and Somnofy [n=17].

## Analysis Period – Manual [AP-M]

**Figure S6. Agreement Matrices for the sleep summary and epoch by epoch (EBE) concordance.** The agreement matrices are represented as heatmaps to illustrate level of concordance with polysomnography (gold standard) derived metrics. Each sleep measure row is colour coded using hot colormap to denote low (dark) to high (light) performance for **a.** symmetric mean absolute percentage error (SMAPE) and **b.** standardized absolute difference (SAD). The sleep summary measures are computed over the Analysis Period - Manual [AP-M]. For the sleep stage agreement, **c.** Matthews correlation coefficient (Matthew’s CC) is used. The number of participants contributing to each of the devices are AWS [n=18], WSA [n=35], Emfit [n=16] and Somnofy [n=17]. All the values are rounded to two decimal places. Unavailability of the measure is indicated using grey. The colour code of all the agreement matrices is scaled across each row.

**Figure S7. Scatter plot showcasing the outliers in the Withings sleep analyser data (WSA) AP-A estimates (n=35).** The data point in red is from a participant with severe arrythmia.
